# Computational identification of antigen-specific T cell groups through generative epitope modeling

**DOI:** 10.1016/j.isci.2026.116505

**Published:** 2026-06-29

**Authors:** Minuk Ma, Wilson Tu, Carlos Vasquez-Rios, Jiarui Ding

**Affiliations:** 1 Department of Computer Science, University of British Columbia, Vancouver, BC V6T 1Z4, Canada

**Keywords:** Biological sciences

## Abstract

Single-cell T cell receptor (TCR) sequencing enables high-resolution analysis of TCR diversity and clonal expansion. However, inferring antigen specificity for individual TCRs remains challenging and typically requires costly functional assays. We introduce EpitopeGen, a transformer-based model that generates candidate epitope sequences from TCR sequences, enabling population-level identification of T cell groups with shared predicted antigen specificity. EpitopeGen employs a semi-supervised strategy that searches over 70 billion candidate TCR-epitope pairs and incorporates high-confidence binding predictions as pseudo-labels under biologically inspired quality control. Generated epitopes exhibit high predicted binding probabilities, sequence diversity, realistic biochemical properties, and biophysical stability. In cancer datasets, EpitopeGen identifies clonally expanded tumor-infiltrating CD8^+^ T cells enriched for tumor-associated antigen recognition and cytotoxic signatures. In patients with COVID-19, it reveals distinct transcriptomic profiles of T cells targeting spike and non-structural proteins across disease severities. EpitopeGen provides a scalable framework for studying disease-associated T cell populations, complementing experimental antigen validation.

## Highlights

• EpitopeGen, trained using semi-supervised learning, generates epitopes from TCRs
• Epitopes recapitulate natural binding, biochemical, and antigen category patterns
• Identifies tumor-reactive CD8^+^ T cells with enhanced cytotoxic signatures
• Distinguishes COVID-19 severity by CD8^+^ T cell antigen recognition profiles

## Graphical abstract

**Figure:**
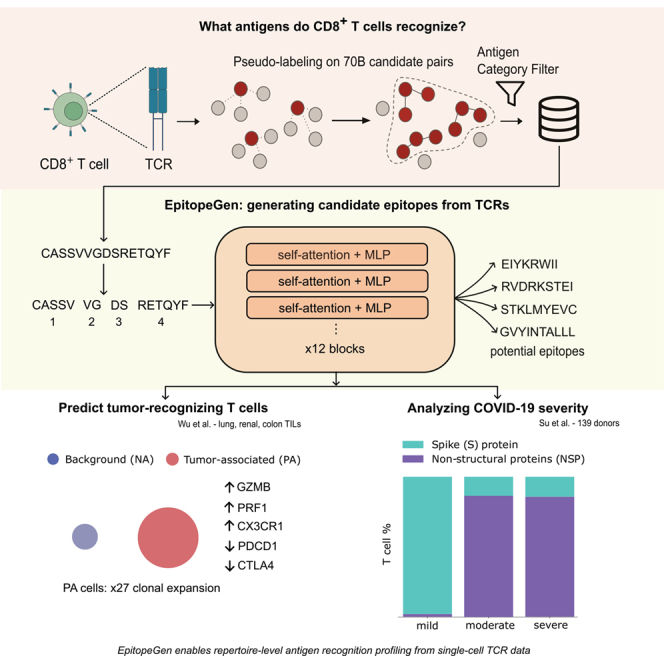

## Teaser

TCR-sequencing, TCR-peptide interaction, sequence-to-sequence generation, semi-supervised learning, transformer

## Introduction

The adaptive immune system provides long-lasting protection against pathogens through T cell receptors (TCRs) that recognize specific antigens. CD8^+^ T cells inspect endogenous peptides displayed on class I major histocompatibility complex (MHC) molecules and eliminate infected cells upon the recognition of abnormal peptides.^1^^,^^2^ The complementarity determining region 3 (CDR3) of the TCR is primarily responsible for epitope binding, with extreme polymorphism resulting from VDJ recombination.^3^^,^^4^ The diversity of the sequence in this region enables diverse immune responses, but presents challenges for quantitative modeling.

Prior computational approaches for TCR analysis include diversity metrics,^5^^,^^6^^,^^7^ clustering methods,^8^^,^^9^^,^^10^ and binding affinity prediction models.^11^^,^^12^^,^^13^^,^^14^^,^^15^^,^^16^^,^^17^^,^^18^^,^^19^ Clustering methods group potentially similar sequences but cannot predict putative epitopes, and may group TCRs with different antigen specificity. Binding affinity predictors face fundamental limitations when applied to repertoire-scale: They can only evaluate pre-selected epitope sets, requiring exhaustive searches across candidate peptides that may miss true high-affinity targets. Static databases may miss novel antigens, and comprehensive cross-reactivity analysis becomes computationally prohibitive due to pairwise comparisons. More critically, existing predictors lack distributional constraints on their outputs. Without such constraints, predictions may be dominated by dataset-biased epitopes, yielding epitope distributions that deviate from expected patterns of CD8^+^ T cell recognition across viral, bacterial, and tumor-associated antigens.

Generative modeling addresses these limitations by directly producing likely epitope targets for each TCR, enabling unbiased repertoire analysis without pre-defined epitope sets. This approach facilitates novel epitope discovery, systematic cross-reactivity analysis, and provides a natural framework for understanding antigenic landscapes recognized by T cell repertoires. Such capabilities could advance precision cancer therapies^20^ and customized vaccine design.^21^

We introduce EpitopeGen, the first sequence-to-sequence generative transformer for generating potential epitope sequences conditioned on TCR sequences. Using a decoder-only architecture with self-attention mechanisms, EpitopeGen learns conditional probability distributions of epitope sequences given TCRs. To overcome the limitation of scarce paired data, we propose BINDSEARCH, a semi-supervised learning framework that systematically evaluates over 70 billion unpaired TCR-epitope combinations using state-of-the-art binding affinity predictors. To minimize false positives and ensure biologically plausible epitope distributions, BINDSEARCH includes three rigorous filtering strategies: (1) Confidence thresholding implements ranking-based selection, where only the top-*k* highest-scoring epitopes are retained as pseudo-labels. This dynamic thresholding approach avoids relying on a specific threshold value. (2) Consistency regularization leverages an ensemble across multiple independently trained models. Only epitope candidates that show high-confidence predictions across the entire model ensemble are selected, improving pseudo-label reliability. (3) Antigen category filter (ACF) calibrates training data according to established immunological principles of CD8^+^ T cell recognition,^22^^,^^23^^,^^24^ adjusting epitope ratios from different antigenic sources (viral, tumor, self) to ensure biological plausibility for repertoire-level analysis. Through this extreme selectivity (0.01% of evaluated pairs), BINDSEARCH generates high-confidence pseudo-labeled data that are merged with existing paired datasets to train EpitopeGen.

We evaluate EpitopeGen across multiple dimensions, including binding affinity, chemical properties, and naturalness, with orthogonal validation through molecular dynamics (MD) simulations.^25^ As expected, while perfect prediction at the individual TCR level remains challenging, our repertoire-level approach yields meaningful biological signals by revealing disease-associated T cell populations via aggregate epitope patterns. We apply EpitopeGen to two practical scenarios: (1) identifying tumor-associated CD8^+^ T cells within the tumor microenvironment,^26^^,^^27^ and (2) characterizing COVID-19-associated CD8^+^ T cells in blood. Tumor-associated CD8^+^ T cells in patients with cancer, especially those clonally expanded in both tumor and adjacent normal tissues, exhibit significantly enhanced cytotoxic markers and reduced exhaustion signatures. In COVID-19, EpitopeGen pinpoints antigen-associated T cells that show clonal expansion and clear cytotoxic profiles in patients with mild and moderate symptoms. In contrast, COVID-19-specific T cells in severe patients retain naive and memory phenotypes rather than acquiring cytotoxic effector functions, potentially explaining the impaired viral control in these individuals. These results demonstrate EpitopeGen’s utility for the computational identification of disease-associated T cell populations, complementing experimental approaches for advancing personalized medicine.

## Results

### A large-scale transformer for conditional epitope generation

**Figure 1:**
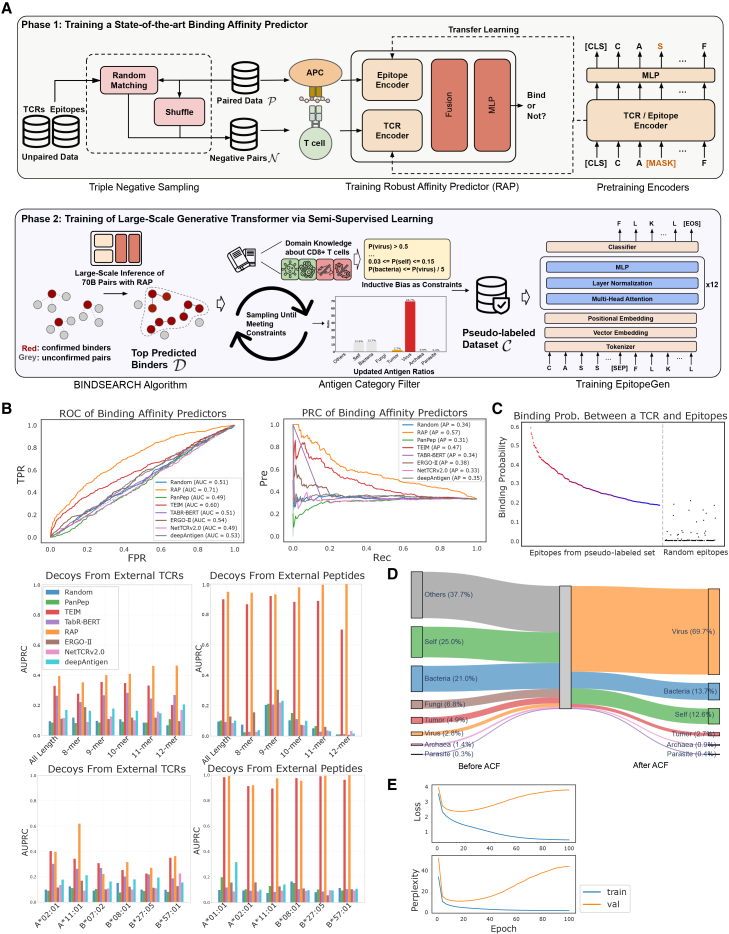
Overview of the EpitopeGen development process based on semi-supervised learning and systematic data balancing (A) Core development process of EpitopeGen. In the first phase, the encoders for TCRs and epitopes are trained based on the masked amino acid prediction task. Triple negative sampling (STAR Methods) was proposed to diversify the sources of negative samples. In the second phase, a large-scale inference was performed to find potential binders. Antigen category filter aligns the antigen source distribution with the reference antigen category distribution through iterative sampling. The resulting pseudo-labeled dataset is used to train a GPT-2 small architecture. (B) Performance of binding affinity predictors on diverse test setups. The bar plots show performance on subsets defined by the peptide length and MHC allele restriction. Negative decoys were prepared from external TCRs or peptides to evaluate realistic generalization scenarios. (C) Binding probability distribution for an example TCR “CAISEGTWETQYF” against epitopes from pseudo-labeled and random datasets. The y axis shows binding probability, while the x axis shows epitope sources. Left shows the binding probability between TCRs and the epitopes from pseudo-labeled data in the decreasing order, and right shows the binding probability between TCRs and arbitrary epitopes. (D) Antigen category distributions in pseudo-labeled datasets before and after the application of the antigen category filter (ACF). Left: Pre-ACF distribution, with many “others,” “self,” and “bacteria” as the dominant categories. Right: Post-ACF distribution, showing a shift to “virus” as the predominant category. “Self” and “tumor” denote self-antigen and tumor-associated antigens, respectively. (E) Learning curves show loss and perplexity by training epoch. See also Figure S1, Tables S1–S5.

To overcome the lack of publicly available data (∼100,000), we propose a semi-supervised learning method, termed BINDSEARCH, which incorporates high-confidence predicted TCR-epitope pairs as pseudo-labels for model training (Figure 1A). As BINDSEARCH requires a reliable binding affinity predictor, we developed the robust affinity predictor (RAP) in the first phase. RAP features a custom BERT^28^ architecture pre-trained on peptide sequences through masked amino acid prediction, followed by an MLP head optimized with the cross-entropy loss. Previous studies generated negative decoys by random shuffling or using external peptides.^11^^,^^16^^,^^19^ We propose triple negative sampling (TNS), which generates decoys using three strategies to reduce overfitting to certain peptide or TCR pools (STAR Methods). RAP showed superior performance compared to Random, PanPep,^12^ TEIM,^16^ TABR-BERT,^17^ ERGO-II,^19^ NetTCRv2.0,^14^ and deepAntigen,^18^ across diverse epitope lengths and MHC alleles (Figure 1B). RAP also showed superior performance when the negative decoys were from external TCRs or peptides, demonstrating high precision. When tested on an external test set^29^ with strictly unseen peptides, RAP, together with TEIM, outperformed other baseline methods (Figure S1).

We collected a pool of 7,331,478 unique TCR sequences from TCRdb.^30^ The 21,801,187 epitope sequences were obtained from three previous publications: NetMHCPan-4.0,^31^ MHCflurry 2.0,^32^ and SysteMHC^33^ (Table S1). In this study, we focus specifically on the binding between CD8^+^ T cells and epitopes displayed on class I MHC molecules, while excluding interactions involving CD4^+^ T cells and epitopes presented by class II MHC molecules. Class I MHC molecules bind and present short epitopes (8–12 amino acids) to CD8^+^ T cells in a restricted manner, which warrants a focused modeling approach.^34^

In the second phase, BINDSEARCH systematically evaluated binding probability across 70 billion unpaired TCR-epitope combinations through a three-stage filtering pipeline designed to minimize false positives while maintaining biological plausibility.

1. Confidence thresholding through relative ranking. For each TCR, BINDSEARCH assessed binding probability against 10,000 randomly sampled epitopes. Rather than applying absolute confidence thresholds, we implemented a relative ranking that selects only the top 2.56% highest-scoring epitopes per TCR as pseudo-positive pairs. This approach accounts for varying baseline prediction distributions across TCRs while maintaining consistent pseudo-label quality. For example, the TCR sequence CAISEGTWETQYF showed a negligible median probability of 0.000 against random epitopes, while the top 256 predicted binders exhibited substantially higher median probability of 0.429 (Figure 1C). This extreme selectivity ensures that only the most confident predictions propagate to training.
2. Consistency regularization via ensemble. BINDSEARCH employs ensemble-based consistency across multiple independently trained models. Only epitope candidates demonstrating consistent high-confidence predictions across the entire ensemble are retained, thereby reducing prediction variance and eliminating model-specific biases. This multi-model validation serves as a critical quality control, ensuring that pseudo-labels represent genuine binding interactions rather than individual model artifacts.
3. ACF for biological plausibility. The intermediate dataset D contained 17 million TCR-epitope pairs (Figure 1A). However, analysis revealed substantial bias, with 37.7% of epitopes categorized as “others” (predominantly eukaryotic sequences). We developed the ACF algorithm, which calibrates antigen distributions according to five fundamental immunological principles of CD8^+^ T cell recognition: (1) viral dominance, (2) limited bacterial presence, (3) endogenous epitope presence, (4) rare fungi and parasites, and (5) absence of pathogenic archaea (STAR Methods). After applying ACF, the resulting curated dataset C (Corpus, ∼700,000) achieved a biologically realistic distribution predominantly consisting of virus-associated epitopes (Figure 1D, Tables S2 and S3).

We developed a custom tokenizer, trained on C, to capture frequently occurring motifs in CDR3*β* and epitope sequences. The model architecture was GPT-2-small, trained for 100 epochs using four NVIDIA L40S GPUs (STAR Methods). The validation loss and perplexity initially decreased smoothly but began to increase after epoch 20 (Figure 1E). For inference, we used top-*k* top-*p* sampling^35^^,^^36^ as the decoding scheme to generate the most probable *k* tokens for a given input TCR, selecting from tokens whose cumulative probability reaches *p* to enhance diversity in the output.

### EpitopeGen generates epitopes with high binding probability, diversity, and balanced species distribution

**Figure 2:**
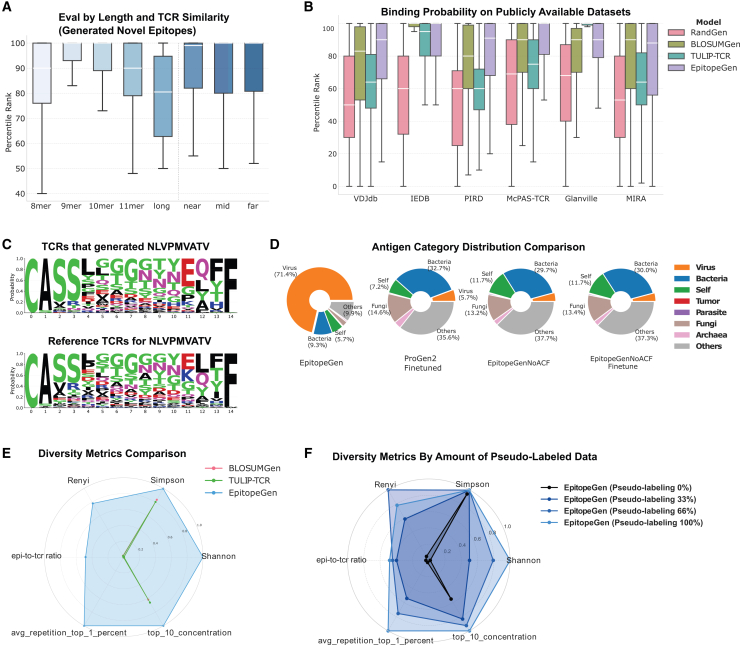
Evaluation of the binding probability, diversity, and distributional characteristics of the generated epitopes (A) Box-and-whisker plots show mean percentile rank (y axis) of binding probability between generated epitopes and query TCRs, focused on unseen epitopes by the epitope length and TCR distance (x axis). Binding probability was computationally measured using TEIM. Long means epitope length >11 amino acids. TCR distance categories are defined by Levenshtein distance to the nearest neighbor in the training set: near (≤1), mid (1 < d ≤ 3), far (>3). (B) Box-and-whisker plots show mean percentile rank (y axis) of binding probability between generated epitopes and query TCR sequences on six test sets (x axis). For each generated epitope, 100 random epitopes were sampled to calculate the percentile rank. Error bars indicate 95% confidence intervals. Binding probability was computationally measured using TEIM. (C) Logomaker plots compare TCRs that generated the epitope NLVPMVATV with reference TCRs known to recognize this epitope from the VDJdb dataset. (D) Distributions of antigen categories for epitopes generated by four models: EpitopeGen, ProGen2 Finetuned, EpitopeGenNoACF, and EpitopeGenNoACFFinetune on the 10x dataset. “Self” denotes self-antigens, and “‘tumor” represents tumor-associated antigens. (E and F) Radar plots comparing six diversity indices (STAR Methods) of generated epitopes using: (E) EpitopeGen versus BLOSUMGen and TULIP-TCR; (F) model variants trained with different proportions of pseudo-labeled data (0%, 33%, 66%, and 100%). Metrics include Rényi diversity (α = 2), Shannon diversity, Simpson’s diversity index, epi-to-TCR ratio (unique generated epitopes/unique input TCR), avg_repetition_top_1_percent (mean repetition count among the top 1% most-generated epitopes), and top_10_concentration (proportion of all generations falling within the 10% most-frequent epitopes). For visualization, unbounded metrics were normalized by their maximum values, and inverse values were used for the last two metrics. Analysis based on the 10× dataset. See also Figures S2–S4.

We evaluated the binding probability, computationally using an external model (TEIM^16^), between the input TCRs and the generated epitopes across multiple test scenarios. A major challenge in this field is to develop a model that generalizes to unseen epitopes. The unseen generated epitopes showed high binding probability regardless of their length (Figure 2A). Also, when measured by the distance between the query TCR and the training set’s TCRs, the epitopes also showed high binding probability (Figure 2A).

Next, we evaluated EpitopeGen on the public test sets: VDJdb,^37^ IEDB,^38^ PIRD,^39^ and McPAS-TCR^40^ (Table S4). Three baseline methods were compared: RandGen, which generates random amino acid sequences; BLOSUMGen, which identifies nearest neighbors using the BLOSUM62 substitution matrix; and TULIP-TCR,^41^ which is a multi-encoder/multi-decoder transformer that leverages domain-specific embeddings. EpitopeGen-generated epitopes demonstrated consistently high binding probability across all public test sets (Figure 2B). Validation using independent experimental datasets by Glanville et al.^42^ and Nolan et al.^43^ (MIRA, Table S5) further showed EpitopeGen’s robustness. Leveraging the ability of GPT-2 to produce multiple, slightly varied outputs for a single input, we analyzed the top 1, 5, 10, and 20 generations. EpitopeGen consistently showed higher mean percentile ranks on public test sets compared to the baselines (Figure S2). These show the effectiveness of our proposed architecture and the semi-supervised learning approach.

As sanity checks of EpitopeGen’s generative behavior, we first compared per-token log-likelihoods of validated, generated, and background epitopes. Generated epitopes showed log-likelihoods comparable to experimentally validated ones and substantially higher than background (Figure S3). We further quantified overlap and similarity to the training corpus: Only 12.3% of top-10 generations for the Glanville et al. TCRs overlapped with the training set, and 0.03% overlapped with the synthetic negative pool. The remaining 87.7% of unseen generations showed a mean normalized Levenshtein similarity of 0.849 to their nearest training epitope (∼1–2 amino acid differences), indicating that EpitopeGen generalizes beyond memorization while staying within a biologically plausible sequence space.

We analyzed the TCR motifs associated with NLVPMVATV (the most frequent epitope in VDJdb), comparing TCRs that generated this epitope (root TCRs) against the ground truths using Logomaker.^44^ The root TCRs and the ground truths showed high similarity, showing the characteristic central “LGGGGYE” sequence (Figure 2C). This shows EpitopeGen’s ability to generate epitope sequences from TCRs that are similar to experimentally validated ones.

We further evaluated EpitopeGen’s performance using repertoire-level test sets, specifically the 10× dataset^45^ released by 10× Genomics, Inc., which contains paired RNA and TCR sequencing of CD8^+^ T cells from a healthy donor. This evaluation mirrors real-world scenarios in which EpitopeGen is used to analyze an individual’s entire TCR repertoire. As a comparison, we fine-tuned ProGen2,^46^ a leading protein language model, on the publicly available training set. We also developed EpitopeGenNoACF, which was trained on the intermediate dataset D without applying ACF, thus lacking inductive bias on the distribution of antigen categories. We trained EpitopeGenNoACFFinetune by fine-tuning EpitopeGenNoACF using C to check if fine-tuning could learn the distribution of antigen categories. We evaluated the models in three key aspects: antigen category distribution, epitope diversity, and redundancy in multiple generations (STAR Methods).

EpitopeGen outperformed the other models in all three aspects. The generated epitopes by EpitopeGen predominantly originated from viruses (Figure 2D), with smaller proportions from tumoral, self, and bacterial sources. This distribution aligns with the immunological principles of viral dominance, limited bacterial presence, and endogenous epitope presence. In contrast, the baselines generated approximately 37.7% and 30% of epitopes from “other” (mostly eukaryotic) and “bacteria,” with notably fewer viral antigens. These skewed antigen distributions highlight the importance of careful data balancing through ACF.

We used six diversity indices to measure epitope diversity (STAR Methods). EpitopeGen showed superior diversity, achieving an epitope-to-TCR ratio of approximately 0.5 (Figure 2E). TULIP-TCR and BLOSUMGen generated the same epitope sequences in many cases, which is not desired for discovering new epitopes. In addition, EpitopeGen showed lower redundancy in its top 20 generations compared to TULIP-TCR (Figure S4A).

To prove the effectiveness of BINDSEARCH, we evaluated models trained with different proportions of pseudo-labeled data: 0%, 33%, 66%, and 100%. The Pseudo-labeling 0% model, trained solely on the public paired dataset, showed considerable redundancy, with the top 1% epitopes repeating approximately 2,000 times on average and a top_10_concentration exceeding 0.95 (Figure 2F). In contrast, Pseudo-labeling 100% achieved higher diversity with a top_10_concentration below 0.50. Epitope diversity improved progressively with increasing proportions of pseudo-labeled data. Moreover, redundancy in the top- *k* generations decreased gradually with higher proportions of pseudo-labeled data for training (Figure S4B).

Repertoire-level evaluations demonstrated that only EpitopeGen generates diverse epitopes with high binding probability while maintaining a balanced antigen category ratio. Together, these properties enable the analysis of TCR populations that potentially recognize disease-associated epitopes, even though precision at the individual TCR level is not yet perfect.

### EpitopeGen generates epitopes with realistic biochemical properties

**Figure 3:**
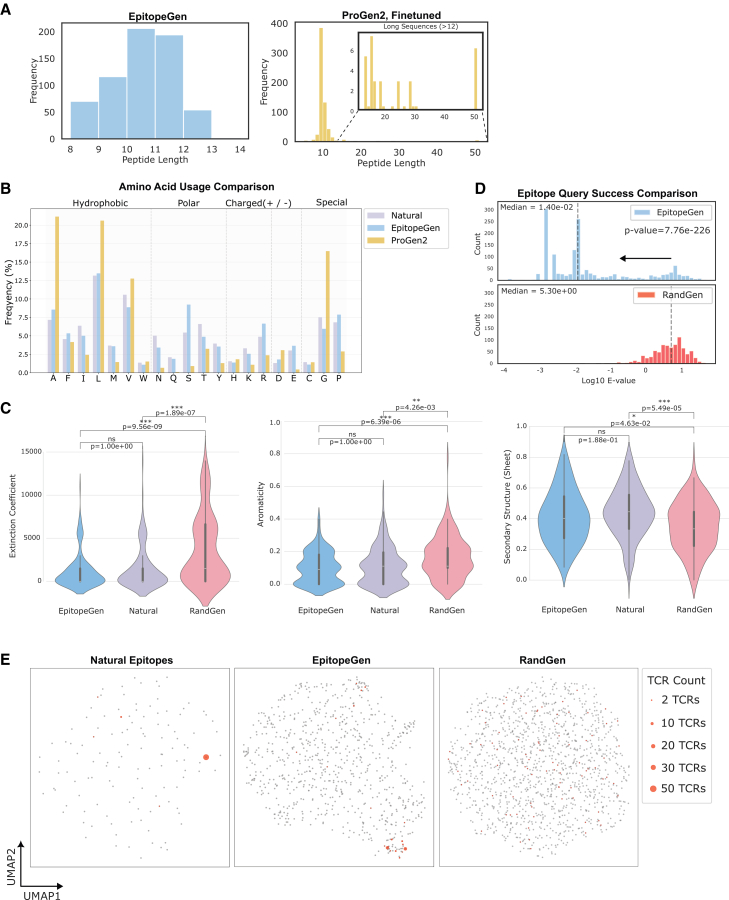
Validation of the naturalness of the epitopes generated by EpitopeGen (A) Length distributions of epitopes that were generated by EpitopeGen and those generated by fine-tuned ProGen2. (B) Comparison of amino acid usage between natural epitopes (natural), EpitopeGen-generated epitopes, and ProGen2-generated epitopes. Amino acids grouped by physicochemical properties: hydrophobic (A, F, I, L, M, V, W); polar uncharged (N, Q, S, T, Y); charged (H, K, R, D, E); special (C, G, P). (C) Distribution of chemical properties for epitopes from EpitopeGen (blue), natural (purple), and RandGen (red). Natural epitopes were sampled from the VDJdb test set (*n* = 642). Chemical properties include extinction coefficient, aromaticity, and secondary structure (sheet). Statistical significance was assessed using two-sided Mann-Whitney *U* tests with Bonferroni correction for multiple comparisons. Each violin plot contains an embedded boxplot displaying the median, interquartile range (IQR), and whiskers extending to 1.5 × IQR. ns means “not significant,” ∗*p* < 0.05, ∗∗*p* < 0.01, and ∗∗∗*p* < 0.001. (D) Distribution of BLASTP E-values for epitopes generated by EpitopeGen (blue, upper) and RandGen (red, lower). Each epitope was queried against the SwissProt DB to identify source proteins. Lower E-values indicate higher statistical significance. Statistical comparison was performed using a one-sided Mann-Whitney *U* test. (E) UMAP visualization of epitope space compares Natural, EpitopeGen, and RandGen epitopes. Epitope similarity was computed using the BLOSUM62 matrix. Each dot represents an epitope, with dot size indicating the number of paired TCRs. Colored dots represent epitopes recognized by TCRs sharing the GLIPH2 motif “CASSIRSQETQYF.” Results are based on the PIRD test set, with natural epitopes sampled from this set. See also Figures S5 and S6.

We next examined the naturalness of the generated epitopes. The generated epitopes had an average length of 10.08, which aligns well with the typical length range^47^ (8–12 amino acids) of the epitopes loaded onto MHC class I molecules (Figure 3A). Additionally, the amino acid usage patterns of the generated epitopes closely resembled those of natural epitopes (Pearson’s correlation = 0.91; Figure 3B).

Recent protein language models, such as ProtGPT2,^48^ ProGen,^49^ and ProGen2,^46^ demonstrated protein sequence generation capabilities but lack precise control mechanisms. Although ProGen can condition input by gene ontology keywords,^50^ adapting these models for specific tasks such as T cell epitope generation remains non-trivial. For comparison, we fine-tuned ProGen2 on public training data. The results show that the fine-tuned model often generated epitopes exceeding the biological length constraints, exhibiting a long-tailed distribution (Figure 3A). This stems from ProGen2’s pre-training on general protein sequences. Specifically, 4.41% of the generated sequences were longer than 12, while 1.47% were shorter than 8 amino acids. The model also showed different amino acid usage patterns compared to natural epitopes (Figure 3B). When tested on the 10× dataset, the fine-tuned ProGen2 showed severe bias, with 83.80% of generated sequences starting with “G.” These findings suggest that T cell epitope generation requires stronger supervision through carefully curated training data that satisfy biological constraints.

To assess the chemical plausibility of the generated epitopes, we analyzed several key properties (STAR Methods) using the ProtParam package.^51^ The distributions of these chemical properties in EpitopeGen-generated epitopes closely mirrored those of natural epitopes, while randomly generated epitopes showed significantly different distributions (Figure 3C; Figure S5).

The generated epitopes were queried against the SwissProt protein database using blastp^52^ to assess the presence of significant matches (STAR Methods). The significance of a blastp search is quantified by the E-value, which represents the expected number of alignments with a score equal to or larger than the observed one by chance. The generated epitopes showed significantly lower E-values (two-tailed Mann-Whitney *U* test, *p* = 1.23 × 10^−244^) compared to randomly generated epitopes, suggesting a significantly higher likelihood of identification within the database (Figure 3D).

Natural and generated epitopes from the same test sets were qualitatively compared through visualization. Epitope embeddings were computed using pairwise BLOSUM similarity, followed by UMAP dimensionality reduction. Within each UMAP, epitopes paired with TCRs in the same motif group (defined using GLIPH2^8^) were color-highlighted. We observed that similar TCRs (in the same motif group) recognize similar epitopes (aggregated colored dots) in both natural epitopes and EpitopeGen-generated epitopes (Figure 3E, left, middle). The colored dots representing EpitopeGen-generated epitopes showed localized aggregation with slight variation. These observations suggest that EpitopeGen produces diverse epitopes that extend beyond those found in publicly available datasets while preserving the recognition patterns of TCR motif groups. In contrast, the colored dots representing RandGen-generated epitopes appeared widely dispersed (Figure 3E, right, Figure S6).

### EpitopeGen generates biophysically stable epitopes

**Figure 4:**
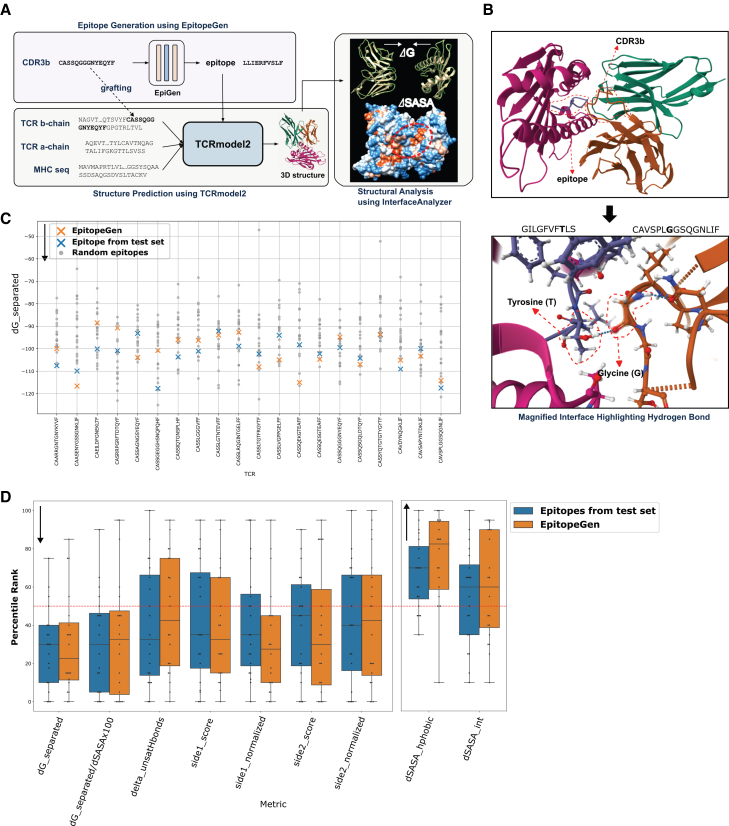
Structural analysis on the TCR-pMHC complexes with the epitopes generated by EpitopeGen using InterfaceAnalyzer (A) A schematic showing the structural analysis. A generated epitope is fed to TCRmodel2, which outputs the 3D structure of the TCR-pMHC complex. This is analyzed using Rosetta Relax and InterfaceAnalyzer, which calculate physicochemical properties. The 3D structures are visualized using Mol∗ Viewer^57^ and Chimera.^58^ (B) Visualization of the predicted 3D structure of a TCR-pMHC complex containing an epitope generated by EpitopeGen. The epitope and CDR3β of the TCR are highlighted. The CDR3β sequence is CAVSPLGGSQGNLIF, and the epitope sequence is GILGFVFTLS. The MHC allele HLA-A∗02:01 is used. The image is a screenshot taken from Mol∗ viewer. The lower image shows the detailed view of the TCR-pMHC interface. A hydrogen bond between the epitope’s tyrosine and the CDR3β′s glycine is highlighted. (C) Binding stability analysis of TCR-pMHC complexes. A scatterplot depicting dG_separated values (y axis), for complexes containing EpitopeGen-generated epitopes (orange) or natural epitopes from the VDJdb test set (blue). dG_separated means the change in Rosetta energy when the interface-forming chains are separated, versus when they are complexed. Analysis is conducted across 20 TCRs (x axis), with 20 random epitopes per TCR serving as controls (gray). Lower dG_separated values indicate higher structural stability in the energy context. dG_separated was calculated using Rosetta’s InterfaceAnalyzer. (D) Boxplots depict the percentile ranks of nine physicochemical properties related to binding affinity for EpitopeGen-generated epitopes (orange) and natural epitopes from the test set (blue). Each dot represents the percentile rank of a property compared to 20 randomly sampled epitopes. The center red line (50%) indicates the performance of a random baseline. Properties were calculated using Rosetta’s InterfaceAnalyzer. Lower values of the first seven properties (dG_separated, dG_separated/dSASAx100, delta_unsatHbonds, side1_score, side1_normalized, side2_score, side2_normalized) and higher values of the last two properties (dSASA_hphobic, dSASA_int) indicate more stable structures or potentially stronger binding. See also Figure S7 and Table S6.

To analyze protein structures and interactions, we use MD simulations, which compute molecular movements over time based on physical and statistical principles.^25^ Specifically, we utilized InterfaceAnalyzer,^53^ from the Rosetta suite,^54^ to estimate TCR-pMHC complex binding affinity. For each CDR3*β*, EpitopeGen generated a corresponding epitope sequence. The complete 3D structure of the TCR-pMHC complex was then constructed using TCRmodel2,^55^ powered by AlphaFold2^56^ (Figure 4A). Due to the high cost of computation, all simulations assumed the prevalent MHC allele HLA-A∗02:01^59^ and utilized a fixed TCR alpha chain (STAR Methods, Table S6). Figure 4B shows an example of the 3D structure (visualized using Mol∗ Viewer^57^ and Chimera^58^) featuring an EpitopeGen-generated epitope. The magnified interface between CDR3*β* (CAVSPLGGSQGNLIF) and the epitope (GILGFVFTLS) highlights a hydrogen bond between tyrosine in the epitope and glycine in the CDR3*β*, contributing to the stability of the complex.

We next evaluated the binding affinity of these complexes using two key metrics. Gibbs free energy (dG_separated) quantifies the energy difference before and after TCR-pMHC binding, providing a measure of binding affinity in an energy context.^60^ Additionally, hydrophobic interactions, crucial forces in protein folding and docking,^61^ were also evaluated. For comparison, we sampled 20 random epitopes from the VDJdb test set as controls. EpitopeGen-generated epitopes showed lower Gibbs free energy compared to randomly sampled epitopes (Figure 4C, orange), with a median percentile rank of 22.5%. This suggests that EpitopeGen-generated epitopes form more energetically stable complexes compared to randomly sampled ones. Furthermore, these epitopes exhibited pronounced hydrophobic interactions (Figure S7), with a mean percentile rank of 82.5%. This observation supports the idea that the generated epitopes form stronger hydrophobic interactions with the CDR3*β* region, potentially burying hydrophobic regions and contributing to binding stability. The natural epitopes from the test set showed a median percentile rank of 30.0% for Gibbs free energy, indicating that they also form more stable TCR-pMHC complexes than random control epitopes.

We analyzed seven additional properties and visualized the aggregated results (Figure 4D). These properties include: binding free energy per unit area (dG_separated/dSASAx100), the number of unsatisfied hydrogen bonds at the interface (delta_unsatHbonds), interface energy scores for TCR and pMHC sides (side1_score and side2_score), normalized side scores (side1_normalized and side2_normalized), and total change in Solvent Accessible Surface Area (dSASA_int).

As a sanity check, epitopes from the test set show mean percentile ranks below 50% on all energy-related properties (dG_separated, dG_separated/dSASAx100, side1_score, side1_normalized, side2_score, and side2_normalized) and delta_unsatHbonds. This aligns with our hypothesis that natural epitopes from the test set form more stable structures compared to randomly sampled epitopes. Notably, EpitopeGen-generated epitopes demonstrated similar trends across all properties, generally showing lower energy-related metrics. In contrast, both natural and generated epitopes displayed higher percentile ranks (≥50%) on properties related to the interaction surface (dSASA_hphobic and dSASA_int). These results suggest that both natural and generated epitopes exhibit stronger interactions at the interface than random epitopes.

These findings indicate that EpitopeGen successfully captures key biophysical characteristics of natural epitopes, particularly energetic stability and hydrophobic interactions.

### EpitopeGen enables the identification of tumor-associated CD8^+^ T cell populations

**Figure 5:**
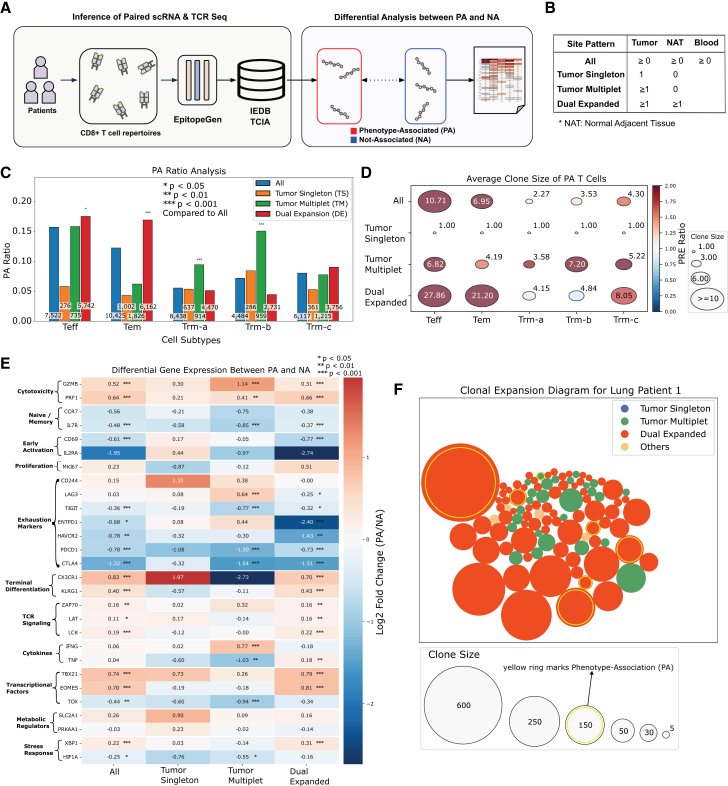
Comparative analysis of PA and NA T cells by different site patterns (A) Schematic of the analysis pipeline. EpitopeGen generates epitopes for T cells, which are queried against the Immune Epitope Database (IEDB)^38^ and The Cancer Immunome Atlas (TCIA)^63^ databases to assess tumor association. TCRs and corresponding epitopes are labeled as phenotype-associated (PA) or not-associated (NA), followed by differential analyses between these groups. (B) Summary of T cell clone patterns across tumor, normal adjacent tissue (NAT), and Blood. Three patterns were defined based on clone size: tumor singleton, tumor multiplet, and dual expanded. An additional category, “all,” was included, in which all cells were considered, regardless of their site patterns. Modified from Wu et al. (C) Proportions of PA TCRs within T cell repertoires, stratified by cell subtype (Teff, Tem, Trm-a, Trm-b, Trm-c) and site pattern (All, tumor singleton, tumor multiplet, dual expanded). One-sided Fisher’s exact tests were performed to test the enrichment of PA T cells in each site pattern to the “‘all” category within each cell subtype. *p* values were adjusted using the Benjamini-Hochberg method (∗*p* < 0.05, ∗∗*p* < 0.01, and ∗∗∗*p* < 0.001). Sample sizes (n) for each site pattern and cell subtype are shown in each bar. Teff: effector T cells; Tem: effector memory T cells; Trm-a/b/c: tissue-resident memory T cell subsets defined by Wu et al.^27^ (D) Mean clone size of PA TCRs by cell subtype (x axis) and site pattern (y axis). Color intensity indicates the phenotype-relative expansion (PRE) ratio, which is the mean clone size of PA T cells divided by that of NA T cells. Red indicates a larger PRE ratio. Circle size represents the mean clone size of PA T cells. We used a one-sided Mann-Whitney *U* test, with *p* values corrected for multiple testing via the Benjamini-Hochberg method. Only significant *p* values are shown. Sample sizes (n) for site patterns [All, tumor singleton, tumor multiplet, dual expanded]: Teff (*n* = 7522, 276, 735, 5742), Tem (*n* = 10425, 1002, 1826, 6162), Trm-a (*n* = 8438, 637, 914, 4470), Trm-b (*n* = 4484, 286, 959, 2731), and Trm-c (*n* = 6117, 361, 1215, 3756). The numbers of PA T cells for site patterns [All, tumor singleton, tumor multiplet, dual expanded]: Teff (1178, 16, 116, 1003), Tem (1272, 43, 113, 1039), Trm-a, (466, 34, 86, 228), Trm-b (321, 24, 144, 121), and Trm-c (490, 19, 94, 338). (E) Heatmap of differential gene expression between PA and NA TCRs, ranked by magnitude of difference. Statistical tests were performed using a two-sided Wilcoxon rank-sum test from Scanpy, and *p* values were adjusted for multiple testing using the Benjamini-Hochberg method (∗*p* < 0.05, ∗∗*p* < 0.01, ∗∗∗*p* < 0.001). Analysis was performed for four site patterns (x axis): All, tumor singleton, tumor multiplet, dual expanded. The curated gene list was obtained from Wu et al. (F) CD8^+^ T cell composition in lung patient 1, combining data from lung tumor tissue, Normal Adjacent Tissue (NAT), and blood data. The top 100 expanded clones are shown, with circle size representing clone size and color denoting cell subtype. PA T cells are marked with yellow rings. See also Figures S8–S10.

We used EpitopeGen to identify CD8^+^ T cells that potentially recognize tumor-associated epitopes. For each TCR, the epitopes are generated and cross-referenced against a combined catalog of tumor-associated epitopes assembled from the Immune Epitope Database (IEDB)^38^ and The Cancer Immunome Atlas (TCIA)^62^ T cells whose generated epitopes matched entries were labeled as phenotype-associated (PA), while those that did not were labeled as not-associated (NA) (Figure 5A). For robustness, we took an ensemble of the predictions by three independently trained EpitopeGen models. We used a dataset published by Wu et al.^27^ that comprises paired single-cell RNA and TCR sequencing from three sites: tumor tissue, normal adjacent tissue (NAT), and peripheral blood. The study identified five CD8^+^ T cell subsets: Teff (effector), Tem (effector memory), Trm-a, Trm-b, and Trm-c (tissue-resident memory cells, with cluster names shortened for brevity). Additionally, the study defined site patterns of TCRs based on their clonal expansion patterns in tumor tissue, NAT, and blood. We focused on three site patterns: tumor singleton (TCR observed only once in tumor tissue), tumor multiplet (TCR observed to be clonally expanded in tumor tissue), and dual expanded (TCR observed at least once in both tumor tissue and NAT) (Figure 5B). “All” means all cells were considered, regardless of their site patterns.

We first analyzed the prevalence of PA T cells (PA ratio) in different cell subtypes and site patterns. PA ratios showed marked heterogeneity by cell subtypes: effector T cells (Teff) showed the highest ratio of 0.157, followed by effector memory T cells (Tem) with 0.122, while three subsets of tissue-resident memory T cells displayed lower ratios (Trm-a: 0.055, Trm-b: 0.072, Trm-c: 0.080) (Figure 5C, blue). Dual expanded cells showed notably elevated PA ratios in Teff and Tem populations (Figure 5C, red), suggesting significant enrichment of effector-like tumor-associated CD8^+^ T cell clones. The tumor multiplet pattern showed elevated PA ratios in Trm-a and Trm-b populations, though at lower levels than Teff and Tem cells. Trm-a and Trm-b cells show lower cytotoxic gene expression compared to Teff and Tem cells,^27^ which suggests potentially diminished anti-tumor efficacy by Tumor Multiplet than by Dual Expanded T cells. Notably, the enrichment of PA T cells within Dual Expanded populations supports the idea of recruitment of tumor antigen-specific CD8^+^ T cells from peripheral sites. Consistent with their lack of clonal expansion, Tumor Singleton T cells showed the lowest PA ratios in Teff, Tem, and Trm-c subtypes.

We next compared the clonal expansion patterns between PA and NA T cells. We define phenotype relative expansion (PRE) ratio as the ratio of average clone sizes between PA and NA TCRs. A PRE ratio of 1.0 indicates equivalent clonal expansion between PA and NA populations. All T cell subtypes showed PRE ratios exceeding 1.0 in the aggregate analysis (Figure 5D, All), which means antigen-specific T cells undergo clonal expansion.^64^ Dual expanded cells (especially Teff and Tem) showed markedly elevated PRE ratios, suggesting coordinated clonal expansion in both tumor tissue and NAT. Tumor multiplet also showed high PRE ratios across all cell subtypes. Patient-specific analysis revealed heterogeneity in clonal expansion patterns (Figure S8). Several patients (lung patient 1, lung patient 2, renal patient 2, and lung patient 4) showed pronounced PRE ratios in dual expanded cells (Figures S8A–S8D). For example, lung patient 1 showed high PRE ratios in Teff, Tem, and Trm-a cells, while renal patient 1 and lung patient 6 showed minimal clonal expansion across all cell subtypes (Figures S8E and S8F). The results reveal heterogeneity of clonal expansion patterns by individuals.^26^

Differential gene expression analysis revealed distinct transcriptional signatures associated with antigen specificity (Figure 5E). In the aggregate analysis (Figure 5E, All), PA T cells showed enhanced cytotoxic potential, by the upregulation of genes encoding cytolytic effector molecules (*GZMB*, *PRF1*), TCR signaling components (*ZAP70*, *LAT*, *LCK*), and effector-associated transcription factors (*TBX21*, *EOMES*), while showing decreased expression of multiple exhaustion markers (*TIGIT*, *ENTPD1*, *HAVCR2*, *PDCD1*, *CTLA4*). A similar signature was observed in Dual Expanded T cells. This profile is similar to that of recently activated effector memory/effector T cells (*T*_EMRA_) described by Zhang et al.,^65^ which showed clonal expansion across blood, normal tissue, and tumor tissue. In contrast, Tumor Multiplet PA T cells showed a mixed exhaustion phenotype (elevated *LAG3* but downregulated *TIGIT*, *PDCD1*, *CTLA4*) and decreased *TNF* expression. These cells lacked the upregulation of TCR signaling markers and key transcription factors. The enhanced activation signatures of Dual Expanded PA T cells may explain the superior response to immune checkpoint inhibitors in patients with those signatures.^27^

We visualized the top 100 clonally expanded CD8^+^ T cells per patient, mapping their spatial distribution, clone size, and phenotype (PA TCRs marked by yellow rings). These visualizations revealed different patterns of T cell repertoire. Lung patient 1 and renal patient 2 showed predominantly Dual Expanded PA TCRs, indicating active replenishment of tumor-recognizing T cells from adjacent tissue (Figure 5F; Figure S9A). In contrast, the repertoire of colon patient 2 consisted mainly of tumor singleton and tumor multiplet T cells (Figure S9B). Lung patient 3 exhibited both patterns associated with the immunotherapy response: clonally expanded PA T cells in dual expanded and tumor multiplet patterns, suggesting complementary anti-tumor mechanisms^27^ (Figure S9C). Lung patient 5 showed numerous dual expanded and tumor multiplet T cells but limited PA T cell expansion (Figure S9D). These varying distributions of tumor-specific and bystander T cells align with previous findings.^66^

Sensitivity analyses of EpitopeGen demonstrated the robustness of our findings across various parameter settings. Specifically, our observations from the tumor dataset remained consistent when varying the number of epitopes generated per TCR (*K*) and the relative proportions of antigen categories in the ACF (Figures S10 and S11).

### EpitopeGen enables the characterization of COVID-19–associated CD8^+^ T cell populations

**Figure 6:**
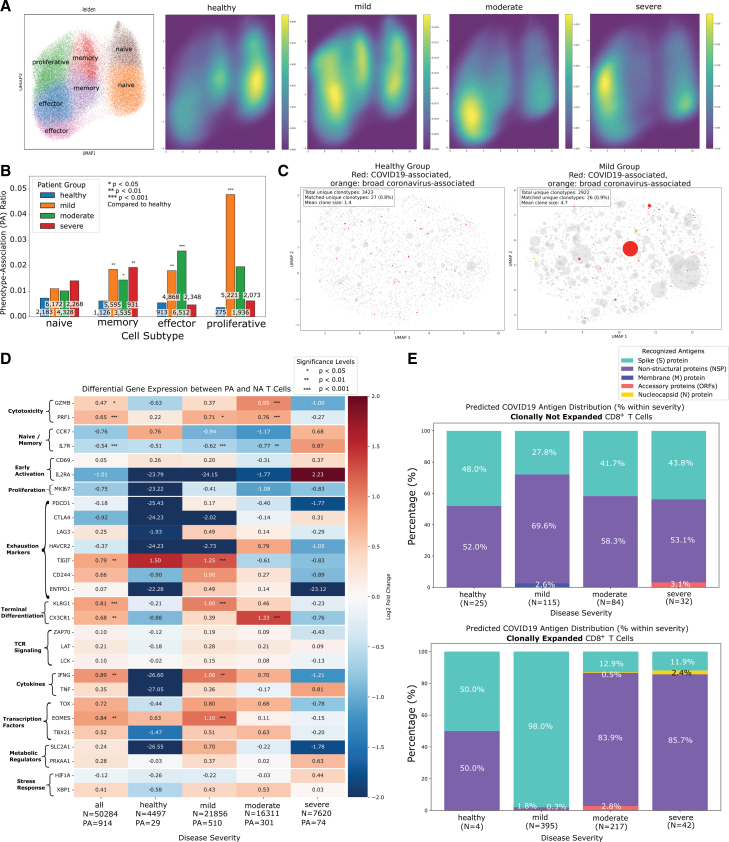
Comparative analysis of PA and NA TCRs from patients with COVID-19 with varying severity (A) UMAP visualization of CD8^+^ T cells based on gene expression data, clustered using the Leiden algorithm (resolution 1.0). Colors represent distinct clusters. Four cell types were identified: naive, memory, effector, and proliferative. Heatmaps were generated using Gaussian kernel density estimation. (B) PA ratio across patient severity groups (healthy, mild, moderate, severe) and T cell subtypes (naive, memory, effector, proliferative). PA ratio is defined as the number of COVID-19- associated TCRs divided by the total TCR count. One-sided Fisher’s exact tests were used within each cell subtype. *p* values were adjusted using the Benjamini-Hochberg method (∗*p* < 0.05, ∗∗*p* < 0.01, and ∗∗∗*p* < 0.001). The sample sizes (n) are shown on each corresponding bar. (C) UMAP visualization of TCRs in healthy and mild groups. Sequence similarity was calculated using the BLOSUM62 substitution matrix. COVID-19-associated TCRs are shown in red, and broad coronavirus-associated TCRs (TCRs whose generated epitopes matched IEDB entries for other human coronaviruses) are in orange. Both plots analyzed the same number of TCRs (4,497). The legend (upper left) displays the total number of clonotypes, COVID-19-associated clonotypes, and the mean clone size. (D) Heatmap shows differential gene expression between PA and NA TCRs, grouped by gene functions. Differential gene expression was assessed using the two-sided Wilcoxon rank-sum test from scanpy, and *p* values were adjusted for multiple testing using the Benjamini-Hochberg method (∗*p* < 0.05, ∗∗*p* < 0.01, and ∗∗∗*p* < 0.001), with log2 fold changes plotted in a color scale. (E) Antigen distribution of PA epitopes across patient groups. The stacked bar plots show antigen proportions for COVID-19 antigens (indicated by distinct colors) in two analyses: (top) proportions based on non-expanded CD8^+^ T cells, and (bottom) proportions based on clonally expanded CD8^+^ T cells. See also Figures S11–S14, Tables S7 and S8.

EpitopeGen was used to identify potential COVID-19-associated CD8 ^+^ T cells using the data from Su et al.^67^ This study provided paired RNA and TCR sequencing data of blood samples from 139 donors, classified into four groups according to the WHO Ordinal Scale (WOS): healthy controls (WOS = 0, no viral infection), mild cases (WOS = 1–2, ambulatory with limited symptoms), moderate cases (WOS = 3–4, hospitalized, potentially requiring supplemental oxygen), and severe cases (WOS = 5–7, hospitalized requiring advanced respiratory support). Seven major cell clusters were grouped into four subtypes based on the gene signatures described by Su et al. (STAR Methods, Figure S12): naive, memory, effector, and proliferative (Figure 6A). Healthy individuals exhibited a predominance of naive cells, while mild cases showed a more balanced distribution of cell subtypes. As the disease severity increased, the T cell populations shifted, with moderate cases dominated by effector cells and severe cases characterized by a prevalence of proliferative cells.

Following the same labeling strategy as in the cancer analysis, candidate epitopes generated for each TCR were cross-referenced against SARS-CoV-2 and broader coronavirus epitopes curated from the IEDB^38^ and Nolan et al.^43^ T cells whose generated epitopes matched any entry were labeled as PA (STAR Methods). We first investigated PA ratios by disease severity. The overall frequency of PA T cells was approximately 1% of the total CD8^+^ T cell population (Figure 6B). Healthy individuals showed consistently low PA ratios in all cell subtypes. In contrast, mild cases—patients who could effectively control COVID-19 without hospitalization—showed elevated PA ratios in memory, effector, and proliferative populations. This suggests that the abundance of antigen-specific T cells enables efficient control of COVID-19.^68^ Similarly, moderate cases maintained high PA ratios in memory and effector populations. However, severe cases showed reduced PA ratios in both effector and proliferative populations, indicating a deficiency in COVID-19-recognizing effector-like T cells (Figure 6B, severe).^69^

We compared the clonal expansion patterns between healthy individuals and mild patients. Each point represents a unique T cell clone where the predicted COVID-19-specific epitopes were colored red, while those associated with broader coronavirus were colored orange to assess potential cross-reactivity (Figure 6C). Although healthy controls showed minimal clonal expansion, mild cases showed pronounced expansion of PA T cells, suggesting antigen-driven clonal expansion.^70^^,^^71^ The average clone size peaked in the mild group, followed by a progressive decrease in the moderate and severe groups. Notably, the severe group showed a smaller average clone size in PA T cells compared to background T cells, suggesting an impaired ability to mount clonal expansion (Figure S13). T cells predicted to recognize broader coronavirus showed a similar severity-dependent expansion pattern. This suggests that both COVID-19-specific and cross-reactive T cell responses may contribute to disease progression.^72^^,^^73^

Next, we analyzed differential gene expression between PA and NA cells by severity group, visualizing log2 fold changes (Figure 6D). In the aggregate analysis, PA T cells showed significantly elevated expression of cytolytic molecules (*GZMB*, *PRF1*), migration markers (*CX3CR1*), antiviral cytokines (*IFNG*), and transcription factors (*EOMES*) (Figure 6D, All), indicating an effector phenotype with active antiviral responses. The presence of exhaustion markers suggested chronic antigenic stimulation in these cells. Mild cases maintained this molecular signature. This phenotype is similar to clonally expanded and effector-like CD8^+^ T cells observed in acute COVID-19 infection associated with improved clinical outcomes.^69^^,^^74^ Notably, PA T cells in severe cases lacked cytotoxic signatures and maintained the expression of naive/memory markers, indicating impaired cytotoxic function, T cell dysfunction, or aberrant differentiation. Our observation details the findings of Su et al.,^67^ who showed that patients with severe symptoms exhibited an increase in naive T cell clusters.

We next analyzed the distributions of the generated epitopes by COVID-19 severity groups. Non-expanded T cell clones maintained balanced recognition profiles between structural and non-structural proteins^75^^,^^76^ across all severity groups, suggesting preserved immune surveillance capacity (Figure 6E, top). In contrast, expanded clones showed marked differences between mild cases and moderate/severe cases. Mild cases exhibited substantial spike protein targeting (98% of expanded clones), while moderate and severe cases displayed a strong skew toward non-structural protein recognition (Figure 6E, bottom). This skew could potentially serve as a predictive marker for disease progression.^77^^,^^78^ The higher proportion of spike-specific T cells in mild cases highlights the importance of structural protein recognition by CD8^+^ T cells in viral control, not only by CD4^+^ T cells, addressing a gap noted in previous studies.^79^^,^^80^^,^^81^ These findings suggest that the proper clonal expansion of CD8^+^ T cells that recognize the spike protein may be crucial for optimal disease control.

Sensitivity analyses showed the robustness of our findings by the number of generations (Figure S14). In addition, we benchmarked EpitopeGen against five established TCR grouping methods on the task of classifying mild versus severe COVID-19 cases via 5-fold subject-level cross-validation (Table S7). When transcriptomic features from PA cells were incorporated, EpitopeGen achieved the highest AUROC (0.762) and AUPRC (0.620) across all methods evaluated (Table S8), indicating that protein-level antigen annotations provide complementary information to sequence-based clustering approaches.

## Discussion

Understanding T cell antigen specificity at the repertoire level is crucial for dissecting human adaptive immunity and developing targeted therapies. While the precise identification of individual TCR-epitope binding pairs remains an aspirational goal, repertoire-level analysis of predicted epitope targets enables practical applications for identifying disease-associated T cell populations. We introduced EpitopeGen, a large-scale generative transformer designed to predict potential epitope sequences from TCR data. To address the fundamental challenge of limited paired TCR-epitope training data, we proposed a semi-supervised learning method that incorporates a large number of unpaired data, complemented by a novel ACF algorithm that ensures biologically plausible antigen category distributions in the training data.

Our comprehensive evaluation showed the necessity of each component in EpitopeGen. Simply training off-the-shelf transformers with public data proved inadequate, resulting in highly redundant epitopes. Similarly, fine-tuning existing protein language models failed to capture natural amino acid distributions. These challenges motivated our key innovations: the RAP for reliable binding prediction, BINDSEARCH for leveraging unpaired data, and the ACF for biologically appropriate antigen distributions. With these components, the generated epitopes satisfied high binding probability, sequence diversity, natural amino acid composition, and biophysical stability. Importantly, when applied to repertoire-scale TCR data, the generated epitopes followed immunologically plausible antigen category distributions.

The application of EpitopeGen to single-cell paired RNA and TCR sequencing datasets revealed important insights into both cancer and COVID-19 contexts. In cancer, PA T cells—especially those clonally expanded in both tumor and normal adjacent tissues—showed enhanced cytotoxic markers and decreased exhaustion marker expression. This supports the hypothesis of continuous peripheral replenishment of tumor-specific T cells.^27^ In COVID-19, PA T cells from mild/moderate patients demonstrated increased clonal expansion with elevated cytotoxic markers compared to healthy individuals, aligning with previously observed activated states.^68^^,^^70^^,^^71^ In contrast, PA T cells in severe patients showed naive/memory markers, suggesting ineffective viral control.^67^ We also identified the clonal expansion of CD8^+^ T cells that recognize broader coronavirus antigens, corroborating previous studies on CD4^+^ T cells.^72^^,^^73^^,^^79^

Beyond cancer and COVID-19, EpitopeGen may be applied to autoimmune conditions where CD8^+^ T cells are pivotal, such as type 1 diabetes^82^ and multiple sclerosis.^83^ Furthermore, EpitopeGen could facilitate a deeper exploration of tumor microenvironments, potentially uncovering novel biomarkers to advance immunotherapy development.

Future work incorporating larger paired datasets, MHC information, and improved binding predictors is expected to enhance prediction accuracy at the individual TCR level.

### Limitations of the study

Several important limitations should be noted. First, EpitopeGen’s predictions are most reliable for repertoire-level comparative analysis (e.g., comparing antigen-specific T cell frequencies across conditions) rather than definitive individual TCR-epitope assignment. The accuracy of individual predictions is constrained by the limited availability and coverage of paired TCR-pMHC training data. Second, the absolute values of PA ratios require careful interpretation due to their dependence on the number of generated epitopes for each TCR, the reference DB, and TCR cross-reactivity. Third, as our current version does not include the MHC information, users may need to check the presentation of the generated epitopes depending on the context. Fourth, the ratios used in ACF may need to be revisited for different applications, such as autoimmune disease or chronic infection. Fifth, while RAP showed superior performance, the limited size of the current training set may have caused confirmation bias in the pseudo-labeling process. Community benchmarks such as IMMREP^84^^,^^85^^,^^86^ will be a valuable resource for evaluating future iterations of RAP and EpitopeGen. Future work will incorporate class II MHC-presented peptides, enabling the study of CD4^+^ T cells. Despite these limitations, our cancer and COVID-19 applications demonstrate that repertoire-level epitope generation provides biologically meaningful insights into disease-associated T cell populations.

## Resource availability

### Lead contact

Further information and requests for resources should be directed to and will be fulfilled by the lead contact, Jiarui Ding (jiarui.ding@ubc.ca).

### Materials availability

This study did not generate new unique reagents.

### Data and code availability

• All data used in this study are publicly available. The datasets used are available through their sources in the key resources table.
• All original code has been deposited at https://github.com/Ding-Group/EpitopeGen and is publicly available as of the date of publication.
• Any additional information required to reanalyze the data reported in this paper is available from the lead contact upon request.

## Acknowledgments

This work was supported by a 10.13039/100002806Discovery grant from the Natural Sciences and Engineering Research Council (10.13039/501100000038NSERC) of Canada, and a department startup fund from the 10.13039/501100005247University of British Columbia (to J.D.). J.D. is a Canada Research Chair and is supported by the Canadian Institutes of Health Research through the Canada Research Chair Program. The computational resource is partially supported by the 10.13039/501100000196Canada Foundation for Innovation & John R. Evans Leader Fund (to J.D.). This research was supported in part through the computational resources and services provided by Advanced Research Computing at the 10.13039/501100005247University of British Columbia.

## Author contributions

M.M. developed and implemented the model, conducted experiments, and prepared the manuscript with input from J.D. W.T., and C.V. assisted with data analysis. J.D. supervised the project. All authors reviewed and approved the manuscript.

## Declaration of interests

The authors declare no competing interests.

## Declaration of generative AI and AI-assisted technologies in the writing process

During the preparation of this work, the authors used Claude 3.5 Sonnet to check the grammar. After using this tool or service, the authors reviewed and edited the content as needed and take full responsibility for the content of the publication.

## STAR★Methods

### Key resources table

**Table:** 

REAGENT or RESOURCE	SOURCE	IDENTIFIER
**Deposited data**		

VDJdb	Shugay, M. et al.^37^	https://vdjdb.cdr3.net/
IEDB	Vita, R. et al.^38^	https://github.com/IEDB/TCRMatch
PIRD	Zhang, W. et al.^39^	E-MTAB-11536
McPAS-TCR	Tickotsky, N. et al.^40^	https://github.com/sidhomj/COVID19
External test set 1	Glanville, J. et al.^42^	https://doi.org/10.1038/nature22976
External test set 2	Nolan, S. et al.^43^	https://clients.adaptivebiotech.com/pub/covid-2020
External test set 3	Francis, J. M. et al.^29^	https://doi.org/10.1126/sciimmunol.adu6730
NetMHCpan-4.0 (epitopes)	Jurtz, V. et al.^31^	https://services.healthtech.dtu.dk/suppl/immunology/NetMHCpan-4.0/
MHCflurry 2.0 (epitopes)	O’Donnell, T. J.^32^	https://data.mendeley.com/datasets/zx3kjzc3yx/3
SysteMHC (epitopes)	Huang, X. et al.^33^	https://systemhc.sjtu.edu.cn/
Unpaired TCRs	Chen, S.-Y. et al.^30^	https://guolab.wchscu.cn/TCRdb/
SwissProt DB	Okada, J. et al.^87^	https://ftp.ncbi.nlm.nih.gov/blast/db/swissprot.tar.gz
Cancer patients data	Wu, T. D. et al.^27^	GSE139555
COVID-19 patients data	Su, Y. et al.^67^	E-MTAB-9357

**Software and algorithms**		

EpitopeGen	This paper	https://github.com/Ding-Group/EpitopeGen
Scanpy	Wolf, F. et al.^88^	https://github.com/scverse/scanpy
Scirpy	Sturm, G. et al.^89^	https://github.com/scverse/scirpy
BLASTP	Camacho, C. et al.^52^	https://ftp.ncbi.nlm.nih.gov/blast/executables/blast+/LATEST

### Experimental model and study participant details

Omitted as our study does not involve biological models.

### Method details

#### Robust affinity predictor (RAP) training

Training and test datasets for binding affinity predictors were compiled from seven sources: VDJdb, IEDB, PIRD, McPAS-TCR, Glanville et al.,^42^ Nolan et al.,^43^ and Francis et al.^29^ Data selection criteria included CDR3*β* lengths of 10-20 amino acids and epitope lengths of 8-12 amino acids. Samples containing non-standard amino acids were excluded. For VDJdb, only samples with vdjdb.score > 0 were considered. From Nolan et al.,^43^ we specifically used their Multiplex Identification of Antigen-Specific T-Cell Receptors Assay (MIRA) dataset from which we sampled 2,000 pairs of TCR and epitope by processing peptide-detail-ci.csv of ImmuneCODE-MIRA-Release002.1. The resulting merged dataset comprised 116,057 TCR-epitope pairs, encompassing 98,460 unique TCRs and 1,141 unique epitopes. We randomly split the dataset into training, validation, and test sets with a 7:2:1 ratio.

As another test set, we used a dataset published by 10x Genomics Inc. containing CD8^+^ T cells from a healthy donor.^45^ This dataset serves as a test set to simulate a real-world application scenario, assessing the model’s ability to generate diverse epitopes with a natural antigen distribution.

Robust Affinity Predictor (RAP), our binding affinity predictor for pseudo-labeling, was developed by modifying TABR-BERT with three architectural changes: implementing a Softmax layer in the head architecture, removing MHC-related architectures, and utilizing PyTorch’s CrossEntropyLoss instead of Contrastive loss. We re-trained the BERT model^28^ solely on epitope sequences, a process that took two days using two NVIDIA L40S GPUs.

A key challenge in training TCR-epitope binding predictors is the lack of confirmed non-binding pairs (negative pairs). For this, we developed Triple Negative Sampling (TNS), which generates diverse negative training examples through three complementary strategies. First, we pair known epitopes with TCRs from a large external pool (TCRNegSet, next section). Second, we generate negative samples by pairing known TCRs with epitopes from a large external pool (EpiNegSet, next section). Third, we randomly pair TCRs and epitopes within the dataset, leveraging the assumption that random TCR-epitope pairs are unlikely to bind. The three sources contribute 25%, 25%, and 50% of negative samples per training batch, respectively. The ratio was empirically determined to balance maintaining dataset distributions against introducing external diversity. Negative pairs were regenerated every five epochs to prevent the model from memorizing specific negative samples. TNS helps reduce potential biases arising from relying on any single strategy. The final model combined the predictions of five independently trained models through ensemble averaging.

#### Large-scale unpaired TCR and epitope datasets

Pseudo-labeling involves large-scale inference on randomly paired TCRs and epitopes using RAP, selecting high-affinity samples. We used the TCRdb dataset, which contained 19,701 TCR sequencing measurements as of May 2024, as the unpaired TCR sequence pool. TCRs with lengths between 10-20 amino acids and comprising only standard amino acids were selected, yielding 7,331,478 unique CDR3*β* sequences. These TCR CDR3*β* sequences were split into TCRCandidateSet (6,831,478) for pseudo-labeling and TCRNegSet (500,000) for Triple Negative Sampling.

Epitope sequences were obtained from NetMHCPan-4.0 (Binding Affinity and Eluted Ligands datasets), MHCflurry 2.0, and SysteMHC (per-MHC datasets from https://systemhc.sjtu.edu.cn/download). Epitopes of 8-12 amino acid length, containing only standard amino acids, were included, resulting in 21,801,187 unique sequences. Inhibitory Concentration 50% (IC50) units were converted to binary labels using the formula lambda x: 1 - log(max(x, 0.1), 50000),^90^ with a threshold of 0.42 defining a hit. The epitope dataset was split into EpiCandidateSet (20,000,000) for pseudo-labeling and EpiNegSet (1,801,187) for Triple Negative Sampling.

#### Semi-supervised learning method

The BINDSEARCH algorithm (Figure S15) generates a pseudo-labeled dataset of TCR-epitope pairs. Given sets of unpaired TCR sequences ${\left\{t_{i}\right\}}_{i=1}^{I}$ and epitope sequences ${\left\{p_{j}\right\}}_{j=1}^{J}$, the algorithm uses a binding affinity predictor function *R* to estimate the binding affinity between TCRs and epitopes (*I* = 6,831,478, *J* = 20,000,000 were used). For each unpaired TCR *t*_*i*_, the algorithm randomly samples *β* = 10,000 candidate epitopes from ${\left\{p_{j}\right\}}_{j=1}^{J}$. The binding affinity *a* is computed for each TCR-epitope pair (*t*_*i*_, *p*) using *R*. Subsequently, the top $n_{\max\_\text{tcr}}=32$ pairs with the highest binding affinities are retained for each TCR. The choice $n_{\max\_\text{tcr}}=32$ trades off between TCR cross-reactivity and computational feasibility. To mitigate redundancy in the resulting dataset, a filtering step is applied. Epitopes that occur more than $n_{\max\_\text{epi}}=100$ times are excluded. Without this filter, we observed unique-epitope ratios as low as 0.0064, meaning the same epitopes were paired with many TCRs (Table S3). The value of $n_{\max\_\text{epi}}=100$ was determined based on the ratio of TCR to epitope observed in public datasets (specifically 116,057 epitopes to 1,141 TCR, resulting in a ratio of approximately 102). In the end, we achieved more balanced TCR-to-epitope ratios across dataset splits (Table S2). This process resulted in a pseudo-labeled dataset comprising |D|=16,909,219 TCR-epitope pairs. The algorithm took four days to run on ten NVIDIA L40S GPUs.

#### Antigen category filter

The intermediate dataset D was biased towards Eukaryotic species, a consequence of the peptide collection methods used by NetMHCPan-4.0, MHCflurry 2.0, and SysteMHC. This bias likely reflects research priorities rather than the biological distribution of antigens recognized by CD8^+^ T cells. To correct this discrepancy, we implemented the Antigen Category Filter (ACF) algorithm (Figure S16). ACF takes as input a set of redundancy-removed pseudo-labeled TCR-epitope pairs ${\left\{\left(t_{l},p_{l}\right)\right\}}_{l=1}^{L}$ and a set of antigen categories with their target ratios ${\left\{\left(c_{n},r_{n}\right)\right\}}_{n=1}^{N}$.

To determine the target antigen ratios, we considered five immunological insights: (1) Viral Dominance: Studies indicate that a substantial proportion of CD8^+^ T cells recognize viral antigens. Masopust et al.^22^ showed that approximately 80% of splenic CD8^+^ T cells recognize viral epitopes during peak infection. Moutaftsi et al.^91^ reported that 29.6% of CD8^+^ T cells produced IFN-*γ* in response to vaccinia virus-infected cells, and Addo et al.^92^ observed robust responses of 10,640 spot-forming cells per million in untreated chronically infected individuals. We set the proportion of viral antigens to be P(virus) ≥ 50%. (2) Limited Bacteria: Although certain bacterial species such as *Listeria monocytogenes* and *Mycobacterium tuberculosis* can elicit CD8^+^ T cell responses through intracellular infection,^93^ and even traditionally extracellular pathogens such as *Staphylococcus aureus* can invade intracellular spaces,^94^ their overall contribution is limited compared to viruses. We set bacterial antigens to P(bacteria) ≤ P(virus) × 0.2. (3) Endogenous Presence: Due to thymic negative selection,^95^ CD8^+^ T cells that recognize self- or tumor antigens are less frequent than virus-specific T cells. Rizzuto et al.^24^ confirmed the significantly lower frequency of self/tumor antigen-specific T cells, Nelson et al.^96^ reported that initial self-reactive T cells are exceptionally rare and difficult to detect prior to antigenic boost, and Pittet et al.^23^ estimated that approximately 1 in 2,500 naïve CD8^+^ T cells recognize tumor (melanoma) antigens. We therefore constrained the self-antigen ratio in the range of 0.03-0.15 and the tumor antigen ratio in the range of 0.01-0.05. (4) Rare Fungi and Parasites: Although some fungi like *Histoplasma capsulatum*^97^ can trigger CD8^+^ T cell responses, their frequency is considerably lower, with only a few dozen fungal species causing regular human infections.^98^ This led us to restrict their combined proportion to P(fungi + parasites) ≤ P(virus) × 0.1. (5) No Reported Pathogenic Archaea: Given the absence of documented archaeal pathogens causing human disease,^99^^,^^100^ we kept the proportion of archaeal antigens very low (P(archaea) ≤ 0.01). The target proportions for each category were sampled from the ranges and normalized to sum to one.

The ACF algorithm begins by identifying the species for each epitope using SwissProtDB through blastp queries (-task blastp-short -evalue 20000 -max_target_steps 10), accounting for the short epitope lengths. This step is encapsulated in the SearchCategory function. The NCBI accession numbers were utilized to query proteins and species using the command: blastdbcmd -db ncbi-blast-2.15.0+/db/swissprot -entry_batch output.txt -outfmt “%a %t”. Species lineage determination involved searching for TaxId from NCBI accession numbers and then querying lineage information from TaxId using epost, esummary, xtract, and efetch commands. The resulting XML files were parsed to categorize the species. The categorization followed hierarchical taxonomic rules: species in families ending in ‘viridae’, containing viral markers (e.g., HIV, SARS, coronavirus), or classified as retroviruses or bacteriophages were assigned as viruses; non-viral species were categorized by superkingdom (Bacteria, Archaea, Eukaryota); eukaryotic species were further sub-classified into Fungi (identified by kingdom markers ‘fungi’ or ‘mycota’), Parasites (recognized through specific genera, families, or parasitic phyla), or ‘others’. The keyword sets used for these classifications are provided in Table S9. Species that could not be definitively categorized were labeled as 'others'. All viral epitopes were retained as the pivot species (counted by the CountPivotCategory function).

#### EpitopeGen architecture and training

EpitopeGen is a decoder-only transformer designed to generate epitope sequences while adhering to specific distributional constraints, including epitope diversity and antigen distributions. The architecture is based on GPT-2, with amino acid sequences encoded using a BPETokenizer^101^ re-trained using TCR and epitope sequences. BPE tokenizer was chosen because conserved motifs in CDR3*β* (including the N-terminal patterns such as ‘CASS’) span multiple amino acids, and a learned subword tokenizer captures these biological units more naturally than character-level encoding. Let V be the vocabulary of tokens. At |V|=400, the tokenizer achieved reconstruction ratios of 99.98% for CDR3*β* sequences and 99.97% for epitope sequences, with 92.5% vocabulary utilization across the training corpus (Figure S17). The BPETokenizer was trained using Corpus C. For a TCR-epitope pair (*t*, *p*), the input sequence is tokenized as:

$$x=\left[\text{BPE}\left(t\right);\left[\text{SEP}\right];\text{BPE}\left(p\right);\left[\text{EOS}\right]\right]$$

where BPE(·) denotes the BPE tokenization function, ‘;’ represents concatenation, [SEP] delineates the boundary between TCR and epitope sequences, and [EOS] marks the end of each sequence. The tokenized sequences were processed using positional embeddings, where position-specific vectors are added to the token embeddings to maintain sequence order information.

We used the GPT2-small architecture^63^^,^^102^ which consists of 12 transformer decoder layers, each containing a multi-head self-attention mechanism followed by a position-wise feedforward network. The self-attention mechanism in each layer has 12 attention heads, allowing the model to attend to different aspects of the input sequence simultaneously. Each head projects the input into query, key, and value spaces of dimension 64 (768/12, where 768 is the dimensionality of each token embedding). The position-wise feedforward network in each layer consists of two linear transformations with a Gaussian Error Linear Unit (GELU) activation function, expanding the intermediate representation to dimension 3,072 before projecting back to 768. This architecture, which contains 124 million parameters in total, was implemented using the HuggingFace^103^ and PyTorch^104^ frameworks. The model defines a probability distribution *p*_***θ***_(x) over a sequence of tokens ***x*** of length *n*, which can be factorized as:

$$p_{\theta }\left(x\right)=\prod_{\tau =1}^{n}p_{\theta }\left(x_{\tau }|x_{<\tau }\right)$$

where ***θ*** represents the model parameters, ***x***_*τ*_ is the *τ*-th token in the sequence, and ***x***_<*τ*_ denotes all tokens before *τ*. This autoregressive formulation allows the model to generate epitope sequences token by token, conditioned on the input TCR sequence. The objective function for training is the negative log-likelihood:

$$L\left(\theta \right)=-\sum_{\left(t,p\right)\in C}\log\;p_{\theta }\left(x\right)\text{.}$$

Given the narrow length distribution of the TCR and epitope sequences compared to natural language paragraphs, each batch contained a single TCR-epitope pair. The AdamW optimizer^105^ was used with parameters: initial learning rate (*α* = 1×10^-5^), *β*_1_ = 0.9, *β*_2_ = 0.999, *ϵ* = 1×10^-8^, and weight decay (*λ* = 0.01). Training took four hours for EpitopeGen and four days for EpitopeGenNoACF using four NVIDIA L40S GPUs.

#### Metrics to evaluate generated epitopes

We employed multiple metrics to evaluate epitope diversity. Shannon Diversity quantifies distribution entropy, considering both unique epitopes and their abundances. Rényi and Simpson’s Diversity, which incorporate quadratic terms, reduce sensitivity to rare epitopes. The epi-to-TCR ratio is calculated as the number of unique generated epitopes divided by unique input TCRs. We also measured the average repetition frequency of top 1% epitopes and the proportion of epitopes within the most frequent 10% to assess redundancy.

Chemical feasibility was assessed using the ProtParam package.^51^ We analyzed extinction coefficient to evaluate light-absorbing properties, aromaticity for hydrophobicity and potential TCR interactions, and secondary structure for folding tendencies. Additionally, we examined isoelectric point to understand pH-dependent charge behavior and instability index to assess stability in solution.

#### Molecular dynamics (MD) simulation

MD simulation provides ways to evaluate the generated epitopes, which is orthogonal to measuring binding affinities using Robust Affinity Predictor, a deep learning-based predictor. After generating an epitope, the first step was to predict the 3D structure of the full TCR-pMHC complex. For this task, we employed TCRmodel2, which is based on AlphaFold2.^56^ The model inputs comprised the full TCR alpha chain, the TCR beta chain, the epitope, and the full MHC sequence, with MHC alleles mapped to sequences referring to the IMGT/HLA Database.^106^ The TCR alpha chain was fixed, while the TCR beta chain’s CDR3*β* region was replaced with the input CDR3*β* sequence. As the MHC allele, we used HLA-A∗02:01 due to its high prevalence in human populations. The amino acid sequences for TCRs and MHC are provided in Table S6. Of the five PDB files generated by TCRmodel2, only pdb_0 was used for subsequent analyses.

The test set comprised 20 CDR3*β* sequences randomly sampled from the VDJdb test set, paired with their corresponding ground-truth epitopes, the EpitopeGen-generated epitopes, and 20 randomly selected control epitopes from EpiCandidateSet, yielding 20×20=440 unique TCR-pMHC complexes for analysis. After predicting the 3D structure, structure refinement and interface analysis were performed using Rosetta 3.14 (released March 7, 2024). The Rosetta relax application (main/source/bin/relax.static.linuxgccrelease) was executed with options -relax:default_repeats 5 -nstruct 1 for structure relaxation. Then, interface analysis was performed using Rosetta’s InterfaceAnalyzer (main/source/bin/InterfaceAnalyzer.static.linuxgccrelease) to examine protein-protein interface properties. Nine interface properties were extracted: binding free energy of separated chains (dG_separated), binding energy normalized by interface area (dG_separated / dSASA×100), change in hydrophobic and total solvent-accessible surface area (dSASA_hphobic, dSASA_int), the number of unsatisfied hydrogen bonds (delta_unsatHbonds), and raw and normalized interface energy scores on each side of the interface (side1_score, side1_normalized for TCR; side2_score, side2_normalized for pMHC). Further details are available in the InterfaceAnalyzer documentation (https://docs.rosettacommons.org/docs/latest/application_documentation/analysis/interface-analyzer).

#### Wu et al. dataset preprocessing

The dataset from Wu et al.^27^ was obtained from Gene Expression Omnibus (GEO) with access number GSE139555, containing clustering, cell type annotation, and TCR contig annotation information. We focused on cells from clusters 8.1-Teff, 8.2-Tem, 8.3a-Trm, 8.3b-Trm, epiand 8.3c-Trm, re-labeled as Teff, Tem, Trm-a, Trm-b, and Trm-c for brevity. The dataset comprised samples from 14 donors in four types of cancer (lung, renal, endometrial, and colorectal), collected from tumor tissue, normal adjacent tissue (NAT), and blood whenever available. The preprocessed data, which contain highly variable genes, UMAP coordinates, cell subtypes, and clonotype information, were initially loaded. To integrate the RNA and TCR sequencing data, we used the scirpy package.^89^ We read the TCR data from files with the contig_annotationst.csv suffix using scirpy’s ir.io.read_10_vdj() function. The resulting information was stored in a MultiModalData (MuData) object, combining both RNA and TCR data. For TCR analysis, we performed quality control using scirpy’s ir.pp.index_chains() and ir.tl.chain_qc() functions. We retained cells with chain pairings classified as ‘single pair’, ‘orphan VDJ’, ‘extra VJ’, or ‘extra VDJ’. In cases of ‘extra VDJ’, we selected the first entry. Only productive TRB chains were included for further analysis, as non-productive chains are excluded by scirpy. For each cell, we extracted the TRB locus, amino acid junction (CDR3_aa), and nucleotide junction (CDR3_nt) sequences. This information was then merged with the gene expression data based on cell identifiers, resulting in a dataset of 36,986 cells with matched gene expression and TCR information. This dataset contained 9,166 unique CDR3_aa sequences and 9,284 unique CDR3_nt sequences. To analyze the expression levels of genes of interest, we utilized the raw dataset of barcodes.tsv, genes.tsv, and matrix.mtx files, which were loaded using scanpy’s^88^ sc.read_10x_mtx() function. We obtained the curated list of genes associated with CD8^+^ T cells from Wu et al., including exhaustion markers (e.g., *PDCD1*, *CTLA4*, *LAG3*), activation and proliferation markers (e.g., *CD69*, *IL2RA*, *MKI67*), TCR pathway-related genes (e.g., *ZAP70*, *LAT*, *LCK*), cytokines (e.g., *IFNG*, *TNF*), and various other markers related to T cell function and differentiation. The data were normalized to a total count of 10^4^ per cell and log-transformed. While the clones were defined based on CDR3_nt, EpitopeGen used CDR3_aa to generate epitope sequences.

These epitopes were then queried against a combined database derived from the Immune Epitope Database (IEDB)^38^ and The Cancer Immunome Atlas (TCIA),^62^ focusing on cancer-related linear epitopes restricted by MHC Class I. From IEDB, we searched entries with the following filters: Epitope Structure: Linear Sequence, Include Positive Assays, No B cell assays, MHC Restriction Type: Class I, Host: Homo sapiens (human), and Disease Data: cancer (ID:DOID:162), anus cancer (ID:DOID:14110, anal cancer), bone cancer (ID:DOID:184), Reference Type: Journal Article, and Disease Data: cancer (ID:DOID:162), anus cancer (ID:DOID:14110, anal cancer), bone cancer (ID:DOID:184), lung cancer (ID:DOID:1324), skin cancer (ID:DOID:4159), hematologic cancer (ID:DOID:2531, blood cancer), brain cancer (ID:DOID:1319), cecum carcinoma (ID:DOID:1519, Cecal cancer), cecum cancer (ID:DOID:1521), colon cancer (ID:DOID:219), liver cancer (ID:DOID:3571), kidney cancer (ID:DOID:263, renal cancer), uveal cancer (ID:DOID:3479), breast cancer (ID:DOID:1612), cervical cancer (ID:DOID:4362, cervix cancer), muscle cancer (ID:DOID:4045), ocular cancer (ID:DOID:2174), rectum cancer (ID:DOID:1993, rectal cancer). The exported data contained 339,908 entries (June 2024). From TCIA (https://tcia.at/neoantigens), we exported all neoanti- gens data without filtering, which contained 1,011,037 rows. The merge of two data contained 1,350,946 entries. The epitopes found in these databases were classified as Phenotype-Associated (PA).

Site patterns of TCRs were encoded as three-character codes representing clonal expansion status in tumor tissue, NAT, and peripheral blood, respectively: uppercase letters (T/N/B) denote clonal expansion (observed more than once), lowercase letters (t/n/b) denote single observation, and ‘x’ denotes absence. From these codes, three site patterns were defined: Tumor Singleton (txb, txB, txx), Tumor Multiplet (Txb, TxB, Txx), and Dual Expanded (any code with both T/t and N/n present). The ‘All’ category included all TCRs regardless of site pattern. Statistical analyses, including comparisons between groups, were performed using two-sided Wilcoxon rank-sum test (scanpy^88^).

#### Su et al. dataset preprocessing

For the analysis of COVID-19-related T-cell responses, we utilized the dataset from Su et al.,^67^ which comprises paired single-cell RNA and TCR sequencing data from 139 donors. We processed the data by first combining all the files with the format heathlab_dc_9_17_pbmc_gex_library_XX_X.txt to create a comprehensive AnnData object indexed by cell barcodes. TCR information was extracted from files formatted as heathlab_dc_9_17_pbmc_cd8_tcr_library_XX_X.txt and used to filter the AnnData object, retaining only cells with associated TCR data. The resulting dataset contained 50,284 unique cells, containing 26,170 unique CDR3 amino acid sequences and 26,621 unique CDR3 nucleotide sequences. We annotated the data with patient demographics and WHO Ordinal Scale scores, mapping the latter to four categories: healthy (0), mild (1 or 2), moderate (3 or 4), and severe (5, 6, or 7). For cell type annotation, we performed standard single-cell RNA sequencing analysis, including normalization, log transformation, selection of 1000 highly variable genes supplemented with the signature genes of CD8^+^ T cells, PCA dimensionality reduction (50 components), Leiden clustering (resolution 1.0), and UMAP visualization. This process identified seven major clusters, which we grouped into four subtypes based on selectively high expression of marker genes: naive (*TCF7*, *LEF1*, *SELL*, *CCR7*), memory (*GZMK*), effector (*NKG7*, *CCL4*, *CST7*, *PRF1*, *GZMA*, *GZMB*), and proliferating (proliferation markers such as *MKI67*, *TYMS*).

To identify COVID-19-associated epitopes, we constructed a catalog by merging two sources. From IEDB, we retrieved linear epitopes from SARS-CoV-2 (ID:2697049), with positive assays, MHC Class I restriction, human host, infectious-disease context, peer-reviewed journal articles, and excluding B-cell assays, yielding 1,780 entries. From the MIRA dataset,^43^ we extracted MHC class I epitopes (n=546) and annotated their protein origins by querying each against NCBI's non-redundant protein database using BLASTP. The merged and deduplicated SARS-CoV-2 catalog contained 1,587 unique epitopes, sorted in increasing order of antigen frequency to prioritize structural proteins, which are underrepresented relative to non-structural proteins.

For the broader coronavirus cross-reactivity analysis, a second catalog was constructed by querying IEDB with the same assay/host/MHC filters but expanding the organism scope to include SARS-CoV-2 and 17 additional coronavirus taxa (covering SARS-CoV, MERS-CoV, human seasonal coronaviruses 229E/NL63/HKU1/OC43, and Alpha/Beta/Gamma/Delta coronavirus genera). For entries exceeding 10 amino acids in length, we generated all overlapping 10-mer subsequences via a sliding window.

We then used EpitopeGen to generate epitopes based on the CDR3 amino acid sequences. The matches were defined by a Levenshtein distance ≤ 1 with the first match selected in the cases of multiple matches. This preprocessing pipeline allowed us to integrate transcriptomic, clonotypic, and epitope-specific information.

#### Ensemble framework and sensitivity analyses

To produce robust PA/NA labels across the single-cell analyses, we trained nine EpitopeGen models in total, organized into three independent ensembles (ens_model_1, ens_model_2, ens_model_3), each composed of three component models. Within each ensemble, the three component models were trained on distinct ACF-balanced corpora generated with different random seeds in the Antigen Category Filter, yielding training sets that maintain the prescribed antigen-category ratio ranges but differ in their specific compositions. Each component model independently produces a candidate PA/NA label per TCR, and ensembling was performed at the label level. With a consensus threshold of 0.5, an ensemble labels a TCR as PA when at least two of its three component models agree; a threshold of 1.0 requires unanimous agreement.

For sensitivity analyses on the Wu et al. dataset, we varied *K* (the number of generated epitopes per TCR) across *K* = {1,2,3,4,5,6}, with primary analyses using *K* = 1, and confirmed that elevated PA ratios in Dual Expanded Teff and Tem populations remained consistent across all values (Figure S10). The three ensembles produced consistent results for both clonal-expansion patterns and differential gene-expression signatures (Figure S11). Analogous sensitivity analyses on the Su et al. COVID-19 dataset are shown in Figure S14.

#### Benchmarking against TCR grouping methods

We compared EpitopeGen with five established TCR grouping methods (GLIPH2,^8^ TCRdist3,^9^ DeepTCR,^10^ iSMART,^107^ and Emerson et al.^108^) on the task of classifying mild (WOS 1-2) versus severe (WOS 5-7) COVID-19 cases from the Su et al.^67^ dataset, using 5-fold subject-level stratified cross-validation. The four grouping methods cluster TCR sequences by sequence similarity and identify disease-enriched clusters, whereas EpitopeGen annotates individual TCRs with predicted antigen targets.

Three classification settings were evaluated. Setting 1 (beta-binomial): the phenotype burden (*k*_*i*_, *n*_*i*_) for subject *i* was computed as the number of unique TCRs in the enriched catalog (*k*_*i*_) over total repertoire size (*n*_*i*_), and modeled per Emerson et al.^108^ as *k*_*i*_ | *n*_*i*_, *c*_*i*_ ∼ *BetaBinomial*(*α*_*l*_, *β*_*l*_, *n*_*i*_) with class-specific parameters estimated by maximum marginal likelihood. Setting 2 (logistic regression on grouping features): L1-regularized logistic regression with balanced class weights, where each subject's feature vector contained the fraction of TCRs hitting each non-singleton cluster, standardized to zero mean and unit variance. Setting 3 (logistic regression with transcriptomic features): features in Setting 2 augmented with the mean expression of key genes (*GZMB*, *PRF1*, *IFNG*, *CX3CR1*, *TIGIT*, *CCR7*, *IL7R*, and *EOMES*). For EpitopeGen, protein-level features were defined as follows. For each subject, we count unique TCRs whose top-k predicted epitopes match known SARS-CoV-2 epitopes, stratified by source protein (Spike, Nucleocapsid, Membrane, Envelope, ORF1ab, Other), and compute epitope diversity and Shannon entropy over the epitope distribution.

Clustering characteristics for each method are summarized in Table S7, and classification performance across the three settings in Table S8.

### Quantification and statistical analysis

All statistical analyses were performed in Python (version 3.10.12). Differential gene expression analyses (Figures 5E and 6D) were performed using the two-sided Wilcoxon rank-sum test implemented in Scanpy (version 1.9.8), with *p*-values adjusted for multiple comparisons using the Benjamini-Hochberg method. Enrichment of phenotype-associated (PA) T cells across site patterns (Figure 5C) and disease severity groups (Figure 6B) was assessed using one-sided Fisher’s exact tests, with *p*-values adjusted using the Benjamini-Hochberg method. Clonal expansion comparisons between PA and NA T cells (Figure 5D) were performed using one-sided Mann-Whitney *U* tests, with *p*-values adjusted using the Benjamini-Hochberg method. Chemical property distributions of generated versus natural and random epitopes (Figure 3C) were compared using two-sided Mann-Whitney *U* tests with Bonferroni correction for multiple comparisons. Natural epitopes were sampled from the VDJdb test set (n=642). BLASTP E-value distributions between EpitopeGen-generated and randomly generated epitopes (Figure 3D) were compared using a one-sided Mann-Whitney *U* test (n=642). Statistical significance thresholds are defined as ∗*p* < 0.05, ∗∗*p* < 0.01, and ∗∗∗*p* < 0.001 throughout. Additional details for each analysis are reported in the corresponding figure legends.
