# Supplementary material for: Computational identification of antigen-specific T cell groups through generative epitope modeling
Source: iScience. 2026 Jun 29;29(7):116505. doi: 10.1016/j.isci.2026.116505 (PMC13378025; doi:10.1016/j.isci.2026.116505)
Supplement: Document S1. Figures S1–S17 and Tables S1–S9 [file mmc1.pdf]

iScience, Volume *29*

Supplemental information

Computational identification of antigen-specific T cell groups through generative epitope modeling

Minuk Ma, Wilson Tu, Carlos Vasquez-Rios, and Jiarui Ding

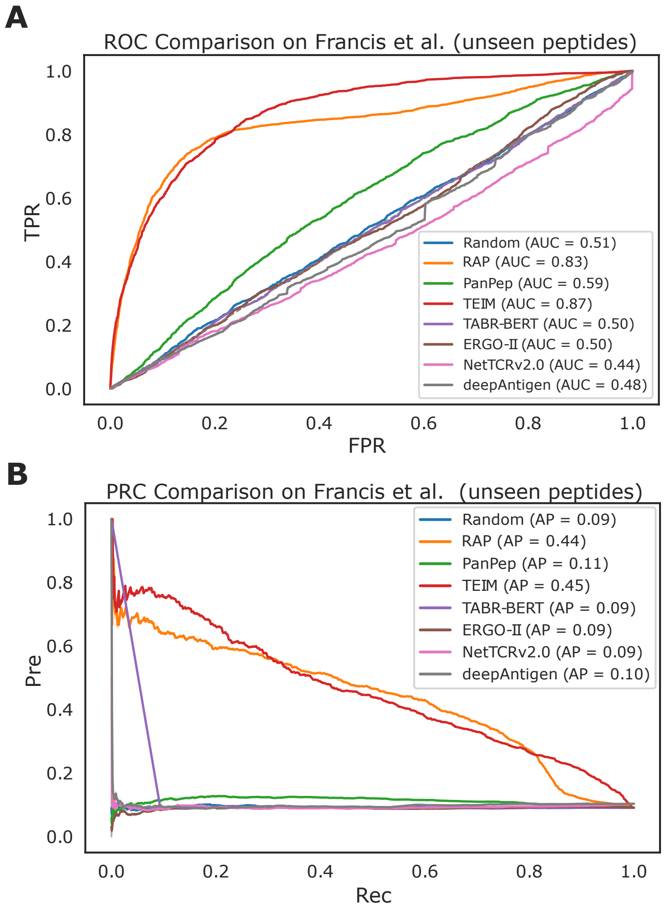

Figure S1: Performance of RAP and other binding affinity predictors on an external dataset, related to Figure 1. **(A)** Receiver Operating Curves (ROC) and **(B)** Precision-Recall Curves (PRC) of Robust Affinity Predictor (RAP) and other competing binding affinity predictors. The test set is from Francis et al.[1] Only unseen peptides that are mutually exclusive to common public datasets (VDJdb[2], IEDB[3], PIRD[4], and McPAS-TCR[5]) were retained. Ten times more negative decoys were generated by pairing TCRs with external peptides. AUC or AP values are denoted in the legends next to the model names.

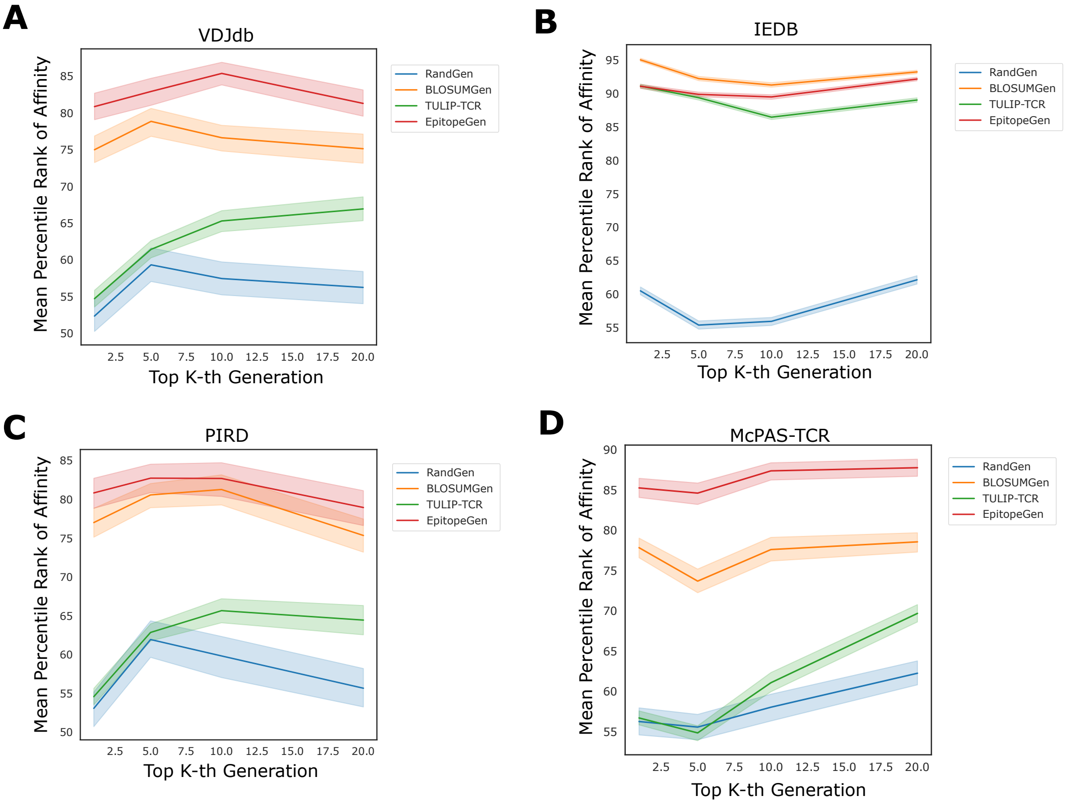

Figure S2: Validation of high binding affinities between EpitopeGen-generated epitopes and input TCRs, related to Figure 2. (**A–D**) The validation results by the test sets: VDJdb, IEDB, PIRD, and McPAS-TCR. The plots show the mean percentile rank of the binding affinities between the top K generated epitopes and TCRs. Four methods were compared: EpitopeGen, TULIP-TCR[6], BLOSUMGen[7], and RandGen.

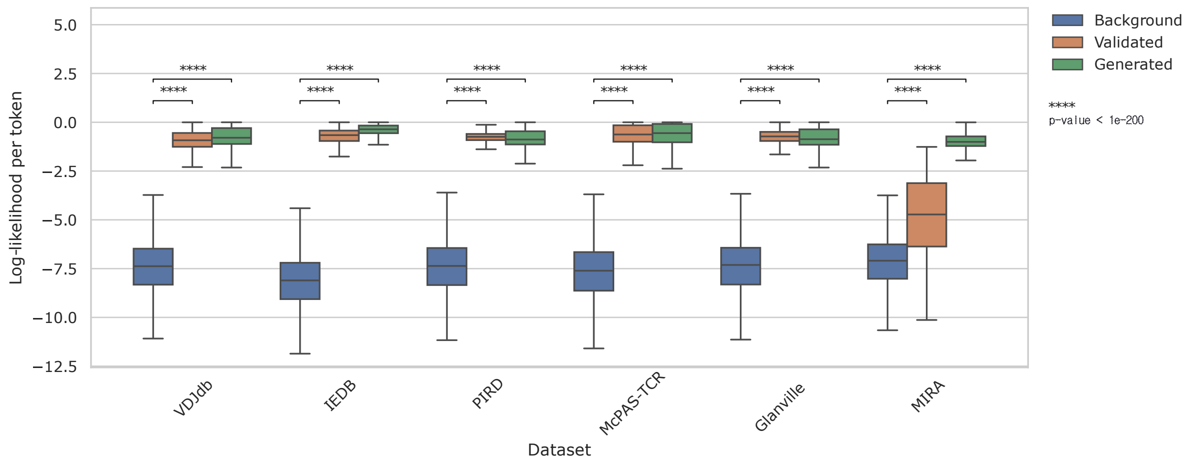

Figure S3: Log-likelihood per token of the validated, generated, and background epitopes, related to Figure 2. Box plots comparing normalized log-likelihood (per token) across three epitope categories: experimentally validated epitopes from each dataset, epitopes generated by EpitopeGen for the corresponding TCRs, and background epitopes. Analysis was performed on four public datasets (VDJdb, IEDB, PIRD, McPAS-TCR) and two external datasets (Glanville et al.[8], MIRA[9]). Validated and generated epitopes show comparable log-likelihoods, both substantially higher than those of background, indicating that EpitopeGen produces sequences with similar statistical properties to experimentally confirmed TCR-binding epitopes. Statistical significance was assessed using one-tailed Mann-Whitney *U* tests.

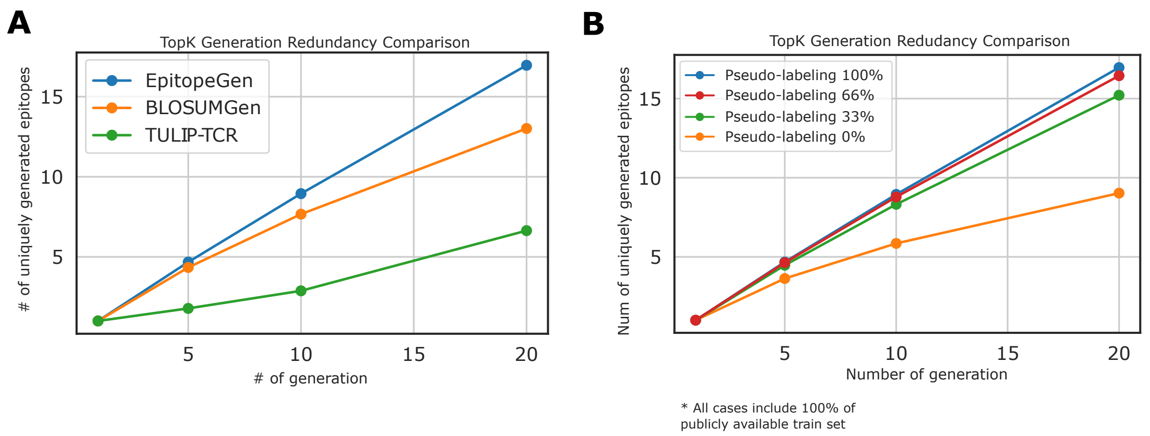

Figure S4: Comparison of redundancy in multiple generations on repertoire-level dataset, related to Figure 2. (**A**) Plot showing the average number of uniquely generated epitopes (y-axis) by the number of generation attempt for each TCR (x-axis). The top generations may produce the same epitope sequences, leading to redundancy. The dataset was the 10x dataset. (**B**) Plot showing the average number of uniquely generated epitopes (y-axis) by the number of generation attempt for each TCR (x-axis). The plot compares four models trained using different amount of pseudo-labeled dataset from 0%, 33%, 66% to 100%. The dataset was the 10x dataset.

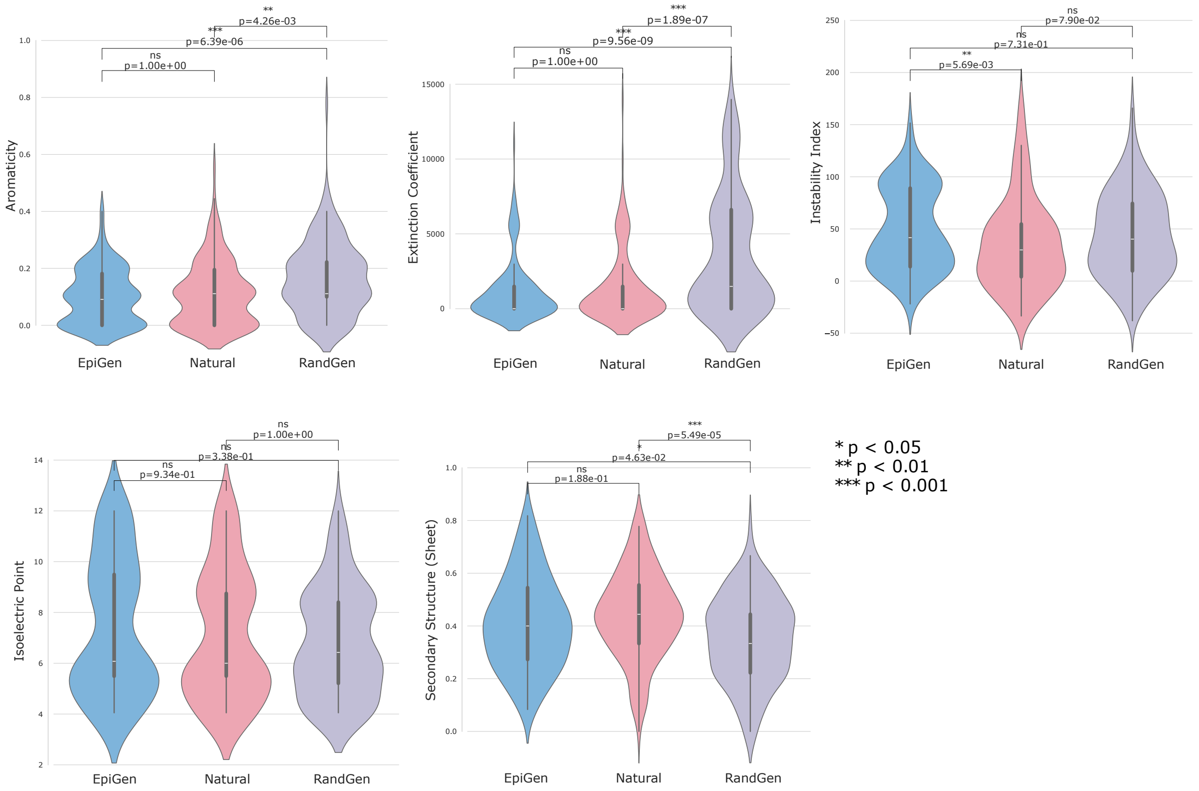

Figure S5: Chemical property distributions comparison, related to Figure 3. Distribution of physicochemical properties across epitope sources, visualized using violin plots. Five properties are analyzed: Aromaticity, Extinction Coefficient, Instability Index, Isoelectric Point, and Secondary Structure (β-sheet content). Epitopes are categorized by source: EpitopeGen-generated (blue), naturally occurring in the test sets (red), and randomly generated (RandGen, purple). The analysis was done using the test set of VDJdb (n=642). Violin plots incorporate box plots showing the median, interquartile range (IQR), and whiskers (1.5 $\times$ IQR). Statistical significance was determined using two-sided Mann-Whitney U tests with Bonferroni correction (*p $<$ 0.05, **p $<$ 0.01, ***p $<$ 0.001, ns: not significant).

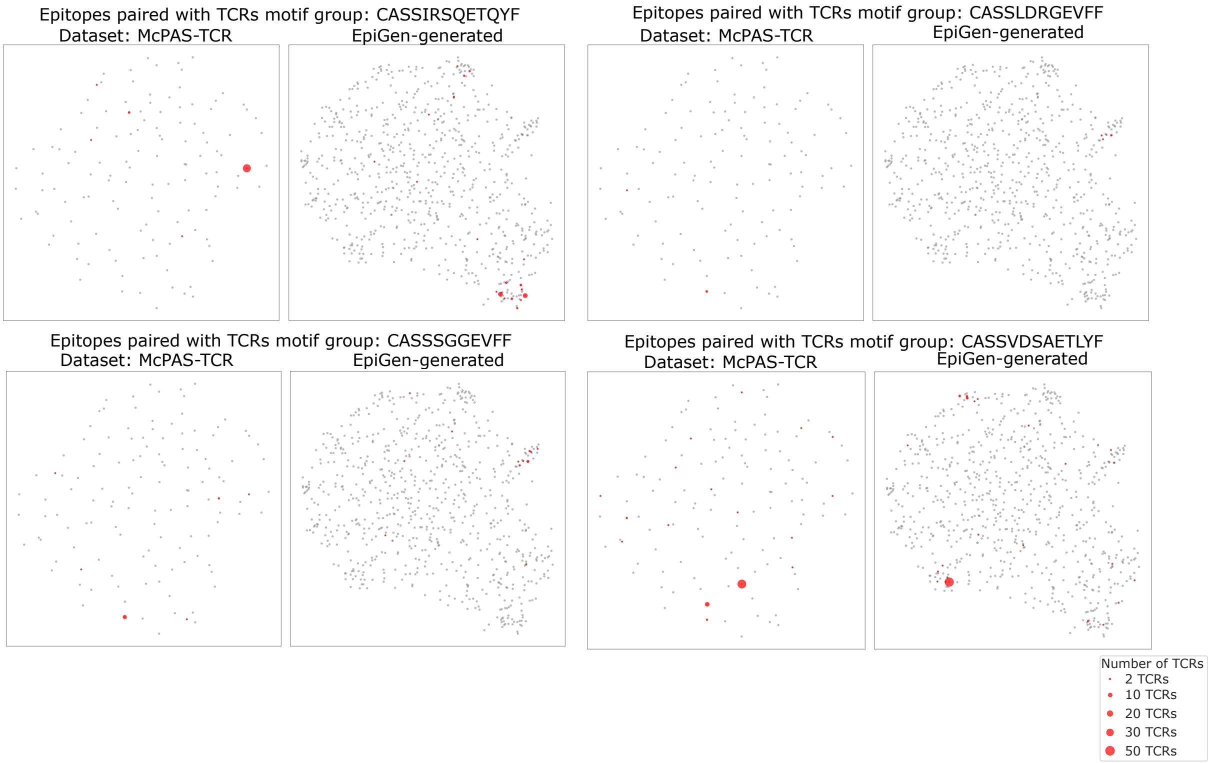

Figure S6: Similar epitopes generated by EpitopeGen tend to be recognized by similar TCRs, related to Figure 3. UMAP visualization of epitope sequence similarity spaces. The dataset used was McPAS-TCR. Sequence similarities were computed using the BLOSUM62 substitution matrix. Each point represents an epitope, with point size proportional to its paired TCR count. Epitopes recognized by TCRs sharing common motifs (identified by GLIPH2[10] analysis of test sets) are highlighted in distinct colors, while other epitopes are shown in grey. Three to four representative TCR motif groups are displayed per dataset. The visualization demonstrates both the broad distribution of generated epitopes and the clustering of similar epitopes recognized by TCRs with shared motifs.

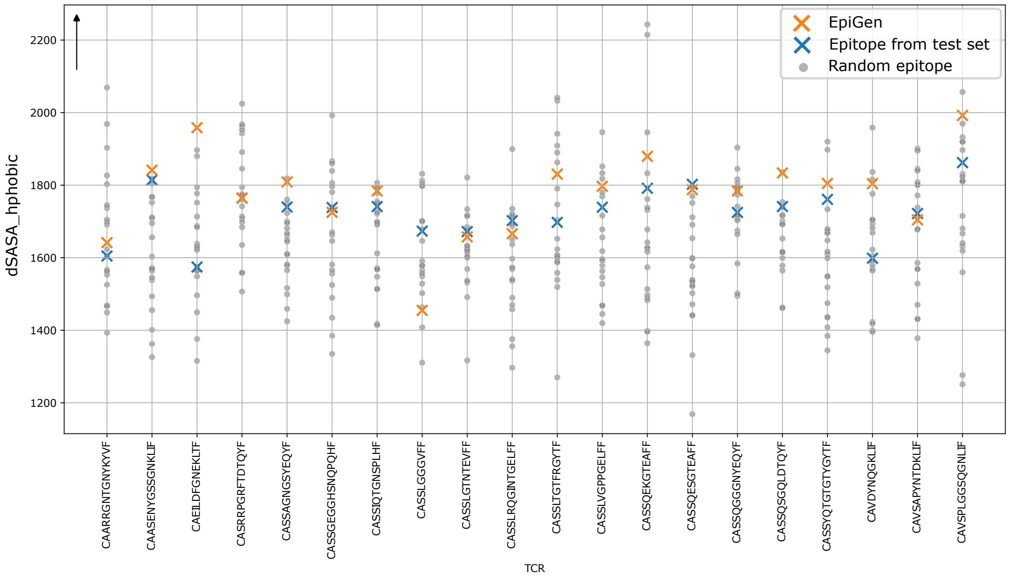

Figure S7: Structural analysis using InterfaceAnalyzer with the generated epitopes, related to Figure 4. Hydrophobic interaction analysis of TCR-pMHC complexes. Scatter plot showing dSASA_hphobic values (y-axis) for complexes containing EpitopeGen-generated epitopes (orange) or natural epitopes from the VDJdb test set (blue). Analysis conducted across 20 TCRs (x-axis), with 20 random epitopes per TCR serving as controls (grey). dSASA_hphobic measures the change in hydrophobic surface area that are hidden inside where dSASA stands for 'delta Solvent Accessible Surface Area'. Higher dSASA_hphobic values indicate higher structural stability. dSASA_hphobic was calculated using `Rosetta`'s `InterfaceAnalyzer`[11,12].

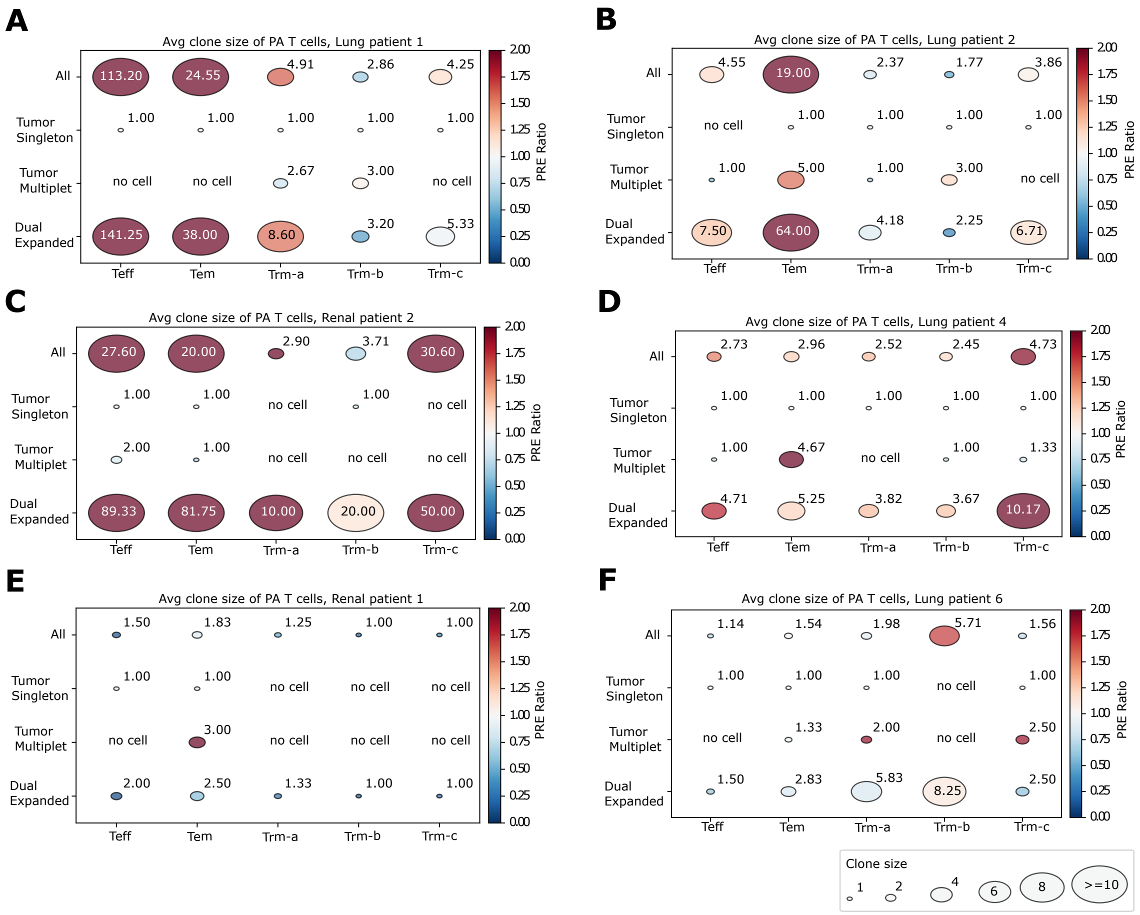

Figure S8: Clonal expansion patterns of Phenotype-Associated T cells across patients, related to Figure 5. (**A**–**F**) Clonal expansion patterns across four site patterns (All, Tumor Singleton, Tumor Multiplet, and Dual Expanded) and five cell subtypes (Teff, Tem, Trm-a, Trm-b, and Trm-c) in six cancer patients (Lung 1, 2, 4, 6; Renal 1, 2). Color intensity indicates the Phenotype-Relative Expansion (PRE) ratio which means the mean clone size of PA T cells divided by that of NA T cells. Red indicates larger PRE ratio. Circle size represents mean clone size of PA T cells. Significant differences between clone sizes of PA and NA T cells were assessed using a one-sided Mann-Whitney U test, with p-values corrected for multiple testing via the Benjamini–Hochberg method. Only significant p-values are shown. “No cell” indicates absence of specific cell populations in that compartment. The plot highlights the heterogeneity of the clonal expansion patterns of PA T cells across patients.

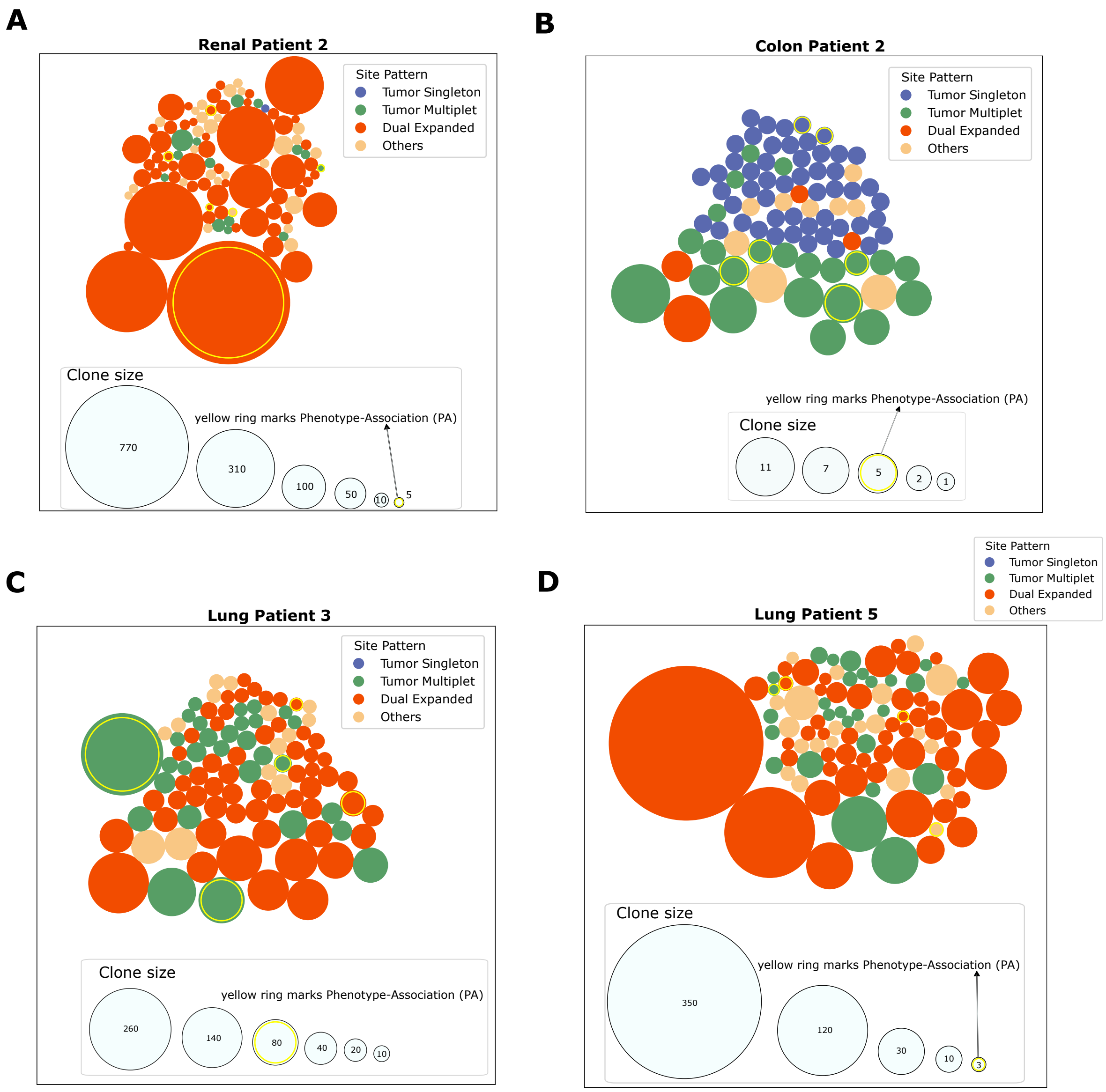

Figure S9: Distribution of clonally expanded T cells in tumor and normal adjacent tissues, related to Figure 5. (**A–D**) Bubble plots illustrating the top 100 clonally expanded T cell clones in four patients: Lung patient 4, Lung patient 3, Endometrium patient 1, and Endometrium patient 3. Two site patterns are highlighted: Tumor Multiplet and Dual Expanded. Circle size represents clone size, while color denotes cell subtype. Phenotype-Associated (PA) T cells are marked by yellow rings. For Tumor Multiplet T cells, the Normal Adjacent Tissue (NAT) region is grayed out, indicating absence of these clones in that area. Dual Expanded T cells are present in both tumor and NAT regions.

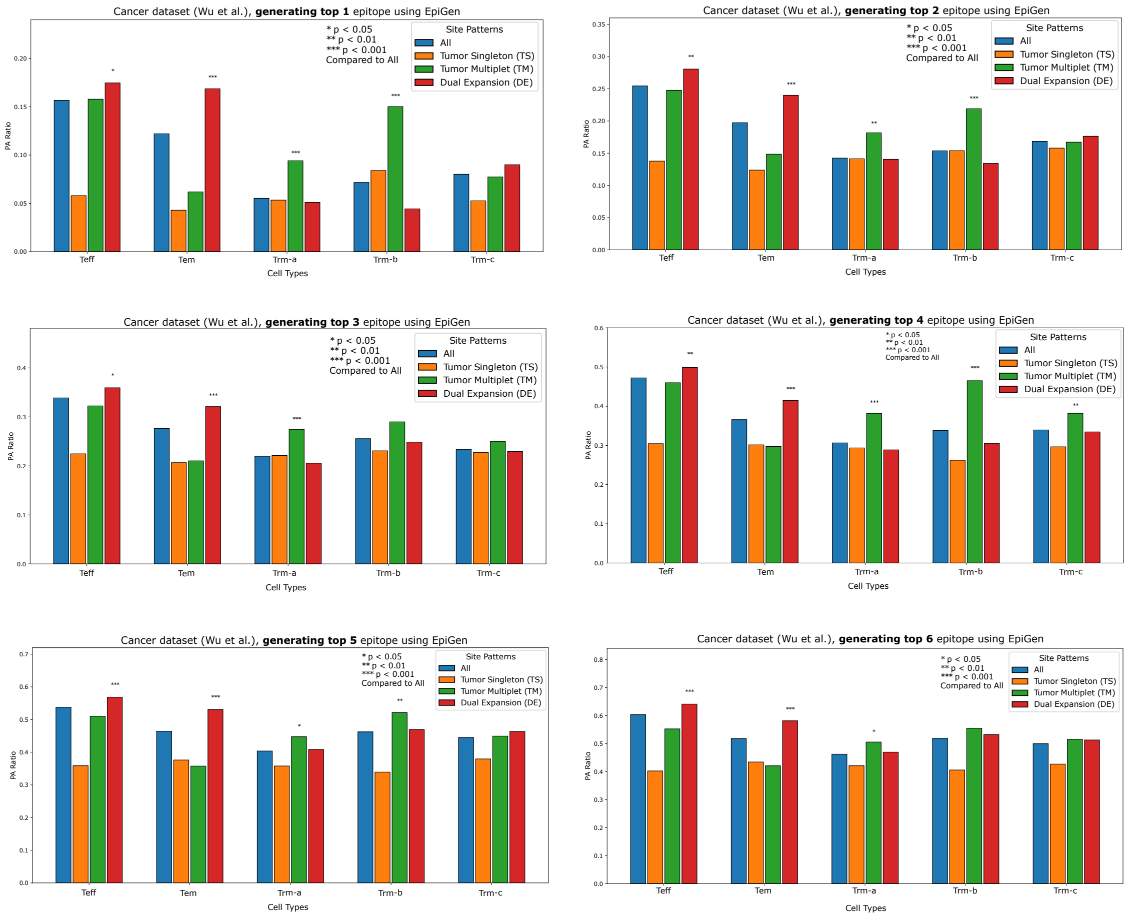

Figure S10: Sensitivity analysis of Phenotype-Association ratios across multiple epitope generation values, related to Figure 5 Analysis of Phenotype-Associated (PA) T cell proportions across different cell subtypes and site patterns as a function of epitope generation count (K). Six values of K (1, 2, 3, 4, 5, and 6) were considered. The PA ratio, defined as the proportion of PA T cells within each TCR repertoire, was calculated for increasing values of K, leveraging EpitopeGen's capability to generate multiple potential epitope sequences per TCR. One-sided Fisher's exact tests were performed to compare PA proportions of each site pattern to the 'All' category within each cell subtype. P-values were adjusted using the Benjamini–Hochberg method (*p $<$ 0.05, **p $<$ 0.01, ***p $<$ 0.001). Sample sizes (n) for site patterns [All, Tumor Singleton, Tumor Multiplet, Dual Expanded]: Teff (n = 7522, 276, 735, 5742), Tem (n = 10425, 1002, 1826, 6162), Trm-a (n = 8438, 637, 914, 4470), Trm-b (n = 4484, 286, 959, 2731), and Trm-c (n = 6117, 361, 1215, 3756). The analysis consistently demonstrates elevated PA ratios in the Dual Expansion pattern in Teff and Tem across different K values, suggesting robust identification of potentially tumor-reactive T cells regardless of generation parameter settings.

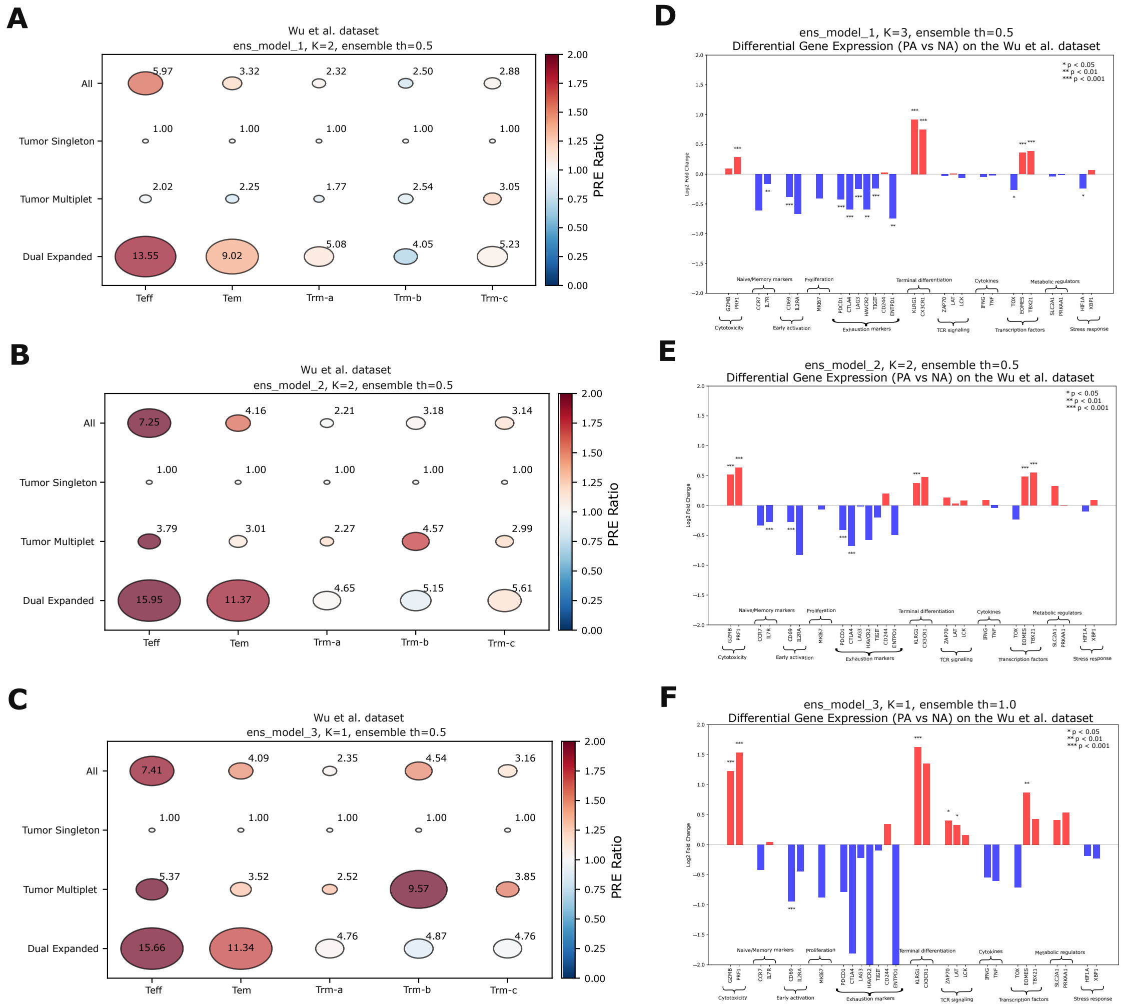

Figure S11: Consistency analysis of three ensemble models on the Wu et al. dataset, related to Figure 5. **a–c.** Clonal expansion analysis across four site patterns (All, Tumor Singleton, Tumor Multiplet, and Dual Expanded) and five T cell subtypes (Teff, Tem, Trm-a, Trm-b, and Trm-c) using three ensemble models: (**a**) `ens_model_1` with generation number $K = 2$, (**b**) `ens_model_2` with $K = 2$, and (**c**) `ens_model_3` with $K = 1$. The ensemble threshold was set to 0.5, labeling a TCR as Phenotype-Associated (PA) if at least two models agreed (average PA label > 0.5). The color scale represents the Phenotype-Relative Expansion Ratio, defined as the average clone size of PA T cells divided by that of NA T cells. Red indicates higher clonal expansion of PA T cells. Circle size and annotated numbers denote the average clone size of PA T cells. **d–f.** Differential gene expression analysis between PA and NA T cells using three ensemble models: (**d**) `ens_model_1` with $K = 3$, (**e**) `ens_model_2` with $K = 2$, and (**f**) `ens_model_3` with $K = 1$. Log_2 fold changes were calculated using the two-sided Wilcoxon rank-sum test from Scanpy, and p-values were adjusted for multiple testing using the Benjamini-Hochberg method.

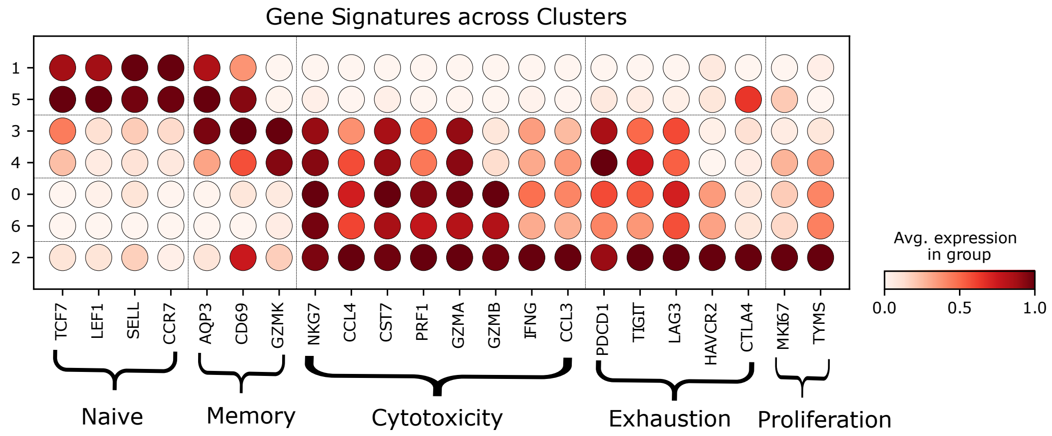

Figure S12: Cluster-specific expression patterns of T cell state-defining gene signatures in COVID-19, related to Figure 6. Systematic characterization of T cell populations by computing the mean expression of signature genes across six distinct clusters identified through dimensionality reduction and unsupervised clustering. The dot plot encompasses five key T cell functional programs: (1) Naive state markers (*TCF7*, *LEF1*, *SELL*, *CCR7*) (2) Cytotoxic effector molecules (*NKG7*, *CCL4*, *CST7*, *PRF1*, *GZMA*, *GZMB*, *IFNG*, *CCL3*) (3) Exhaustion markers (*PDCD1*, *TIGIT*, *LAG3*, *HAVCR2*, *CTLA4*) (4) Proliferation indicators (*MKI67*, *TYMS*) (5) Memory-associated genes (*AQP3*, *CD69*, *GZMK*). Color intensity represents mean expression levels, enabling identification and annotation of distinct T cell states in the context of COVID-19 infection.

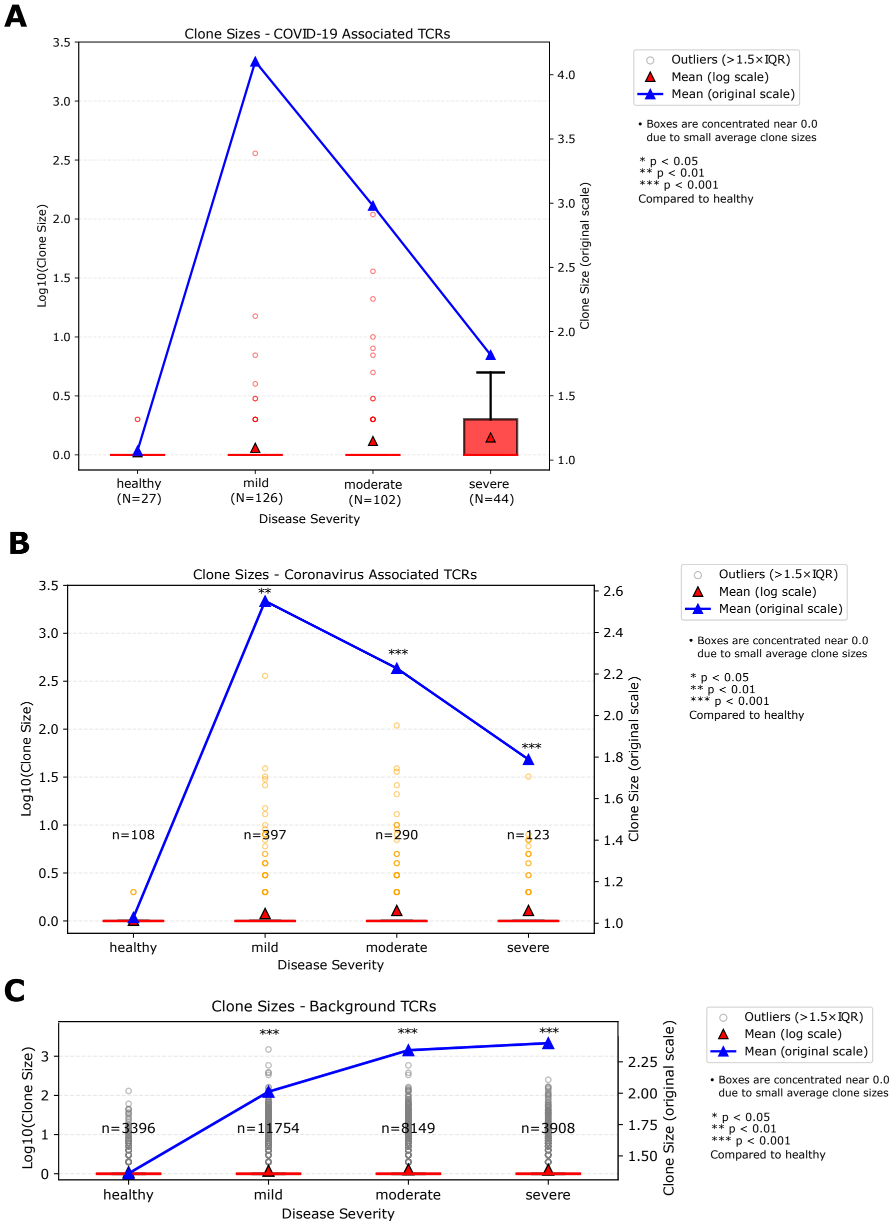

Figure S13: Clonal expansion of COVID-19-Associated, coronavirus-associated, and background T cells by severity group, related to Figure 6. (**A**–**C**) T cell clonal expansion analysis across COVID-19 disease severity (healthy, mild, moderate, and severe) for three T cell categories: (**A**) COVID-19-specific, (**B**) broad coronavirus-associated, and (**C**) non-COVID-19-associated T cells. T cells were labeled based on EpitopeGen-predicted epitopes followed by database querying to determine phenotype associations. Box plots display clone size distribution (left y-axis, log10 scale), with outliers (colored dots) representing significantly expanded clones. Mean clone sizes are indicated by red triangles (log scale, left y-axis) and blue triangles (original scale, right y-axis). Statistically significant differences compared to the healthy group were determined by one-sided Mann-Whitney U tests with p-values corrected using the Benjamini-Hochberg method.

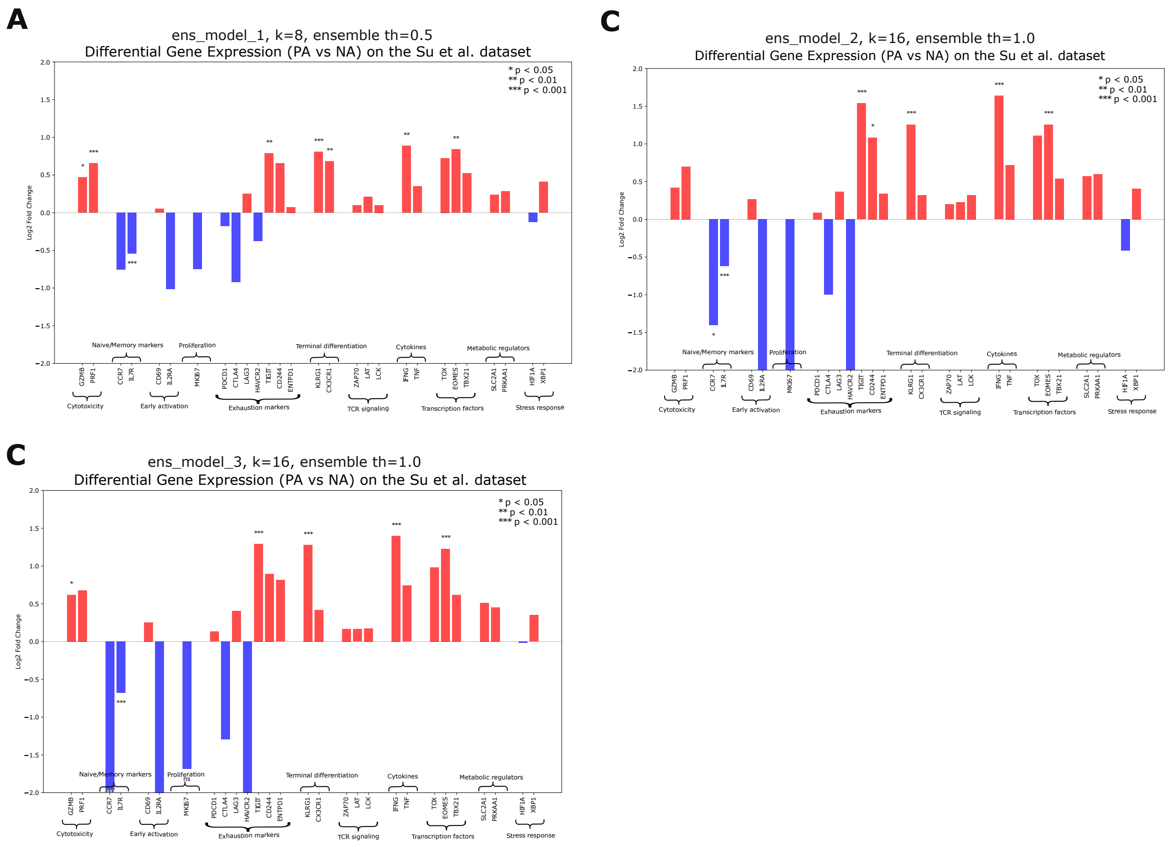

Figure S14: Consistency analysis of three ensemble models on the Su et al. dataset, related to Figure 6. **a–c.** Differential gene expression analysis between Phenotype-Associated (PA) and Not-Associated (NA) T cells using three ensemble models: (**a**) `ens_model_1` with generation number $K = 8$, (**b**) `ens_model_2` with $K = 16$, and (**c**) `ens_model_3` with $K = 16$. The ensemble threshold of 0.5 indicates that a TCR is labeled as PA if at least two models agree, while a threshold of 1.0 requires consensus among all three models. Log_2 fold changes were computed using the two-sided Wilcoxon rank-sum test implemented in Scanpy, and p-values were corrected for multiple testing using the Benjamini-Hochberg method.

Algorithm 1 BINDSEARCH

Require:
$\{t_i\}_{i=1}^{I}$: Set of unpaired TCR sequences
$\{p_j\}_{j=1}^{J}$: Set of unpaired epitope sequences
R: Binding affinity predictor function (RAP)
β: Number of epitopes to check for each TCR (10000)
$n_{\text{max\_tcr}}$: Maximum number of epitopes per TCR (32)
$n_{\text{max\_epi}}$: Maximum occurrences of an epitope (100)
Ensure: $\mathcal{D}$: A dictionary of TCR–epitope pairs with binding affinities

function BINDSEARCH($\{t_i\}_{i=1}^{I}, \{p_j\}_{j=1}^{J}, R, \beta$)
    $\mathcal{D} \leftarrow \{\}$
    for $t_i \in \{t_i\}_{i=1}^{I}$ **do**
        $Q_i \leftarrow RandomSample(\{p_j\}_{j=1}^{J},\ \beta)$
        $A_i \leftarrow \{\}$
        for $p \in Q_i$ **do**
            $a \leftarrow R(t_i, p)$
            $A_i \leftarrow A_i \cup \{(p, a)\}$
        end for
        $\mathcal{D}[t_i] \leftarrow TopK(A_i,\ n_{\text{max\_tcr}})$
    end for
    $\mathcal{D} \leftarrow FilterRedundancy(\mathcal{D},\ n_{\text{max\_epi}})$
    return $\mathcal{D}$
end function

function FilterRedundancy($\mathcal{D},\ n_{\text{max\_epi}}$)
    $C \leftarrow CountEpitopeOccurrences(()\mathcal{D})$
    for $(t_i, \text{pairs}) \in \mathcal{D}.\text{items}()$ **do**
        $\mathcal{D}[t_i] \leftarrow \{(p, a) \in \text{pairs} : C[p] \leq n_{\text{max\_epi}}\}$
    end for
    return $\mathcal{D}$
end function

Figure S15: Algorithm 1. BINDSEARCH, related to STAR Methods The BINDSEARCH algorithm inspects the binding probability of randomly paired TCRs and epitopes and chooses the top candidates.

Algorithm 2 Antigen Category Filter (ACF)

Require:
$\mathcal{D} = \{(t_l, p_l)\}_{l=1}^{L}$: Redundancy-removed pseudo-labeled TCR–epitope pairs
$\{(c_n, r_n)\}_{n=1}^{N}$: Antigen categories and target ratios, with $\sum_{n=1}^{N} r_n = 1$, $N = 9$, and r_1 the ratio for the pivot
Ensure: $\mathcal{C}$: Corpus of TCR–epitope pairs with balanced antigen categories

function AntigenCategoryFilter($\{(t_l, p_l)\}_{l=1}^{L},\ \{(c_n, r_n)\}_{n=1}^{N}$)
  $\{(t_l, p_l, c_l)\}_{l=1}^{L} \leftarrow \textit{SearchCategory}(\{(t_l, p_l)\}_{l=1}^{L})$
  $M \leftarrow \textit{CountPivotCategory}(\{(t_l, p_l, c_l)\}_{l=1}^{L})$
  $\{c'_n\}_{n=1}^{N} \leftarrow \{M \cdot r_n / r_1\}_{n=1}^{N}$ ▷ Target counts per category
  $\mathcal{C} \leftarrow \{\}$
  for $n \in \{1, \dots, N\}$ **do**
    $S_n \leftarrow \{(t_l, p_l) : (t_l, p_l, c_l) \in \{(t_l, p_l, c_l)\}_{l=1}^{L} \text{ and } c_l = c_n\}$
    $\mathcal{C} \leftarrow \mathcal{C} \cup \textit{RandomSample}(S_n,\ c'_n)$
  end for
  return $\mathcal{C}$
end function

function SearchCategory($\{(t_l, p_l)\}_{l=1}^{L}$)
    ▷ Use `blastp` against SwissProt and NCBI taxonomy queries
    ▷ to determine the species and antigen category c_l of each pair
  return $\{(t_l, p_l, c_l)\}_{l=1}^{L}$
end function

function CountPivotCategory($\{(t_l, p_l, c_l)\}_{l=1}^{L}$)
  return $\left|\{(t_l, p_l, c_l) \in \{(t_l, p_l, c_l)\}_{l=1}^{L} : c_l = \text{“Virus”}\}\right|$
end function

Figure S16: Algorithm 2. Antigen Category Filter, related to STAR Methods The Antigen Category Filter maintains biologicall plausible species in the training dataset.

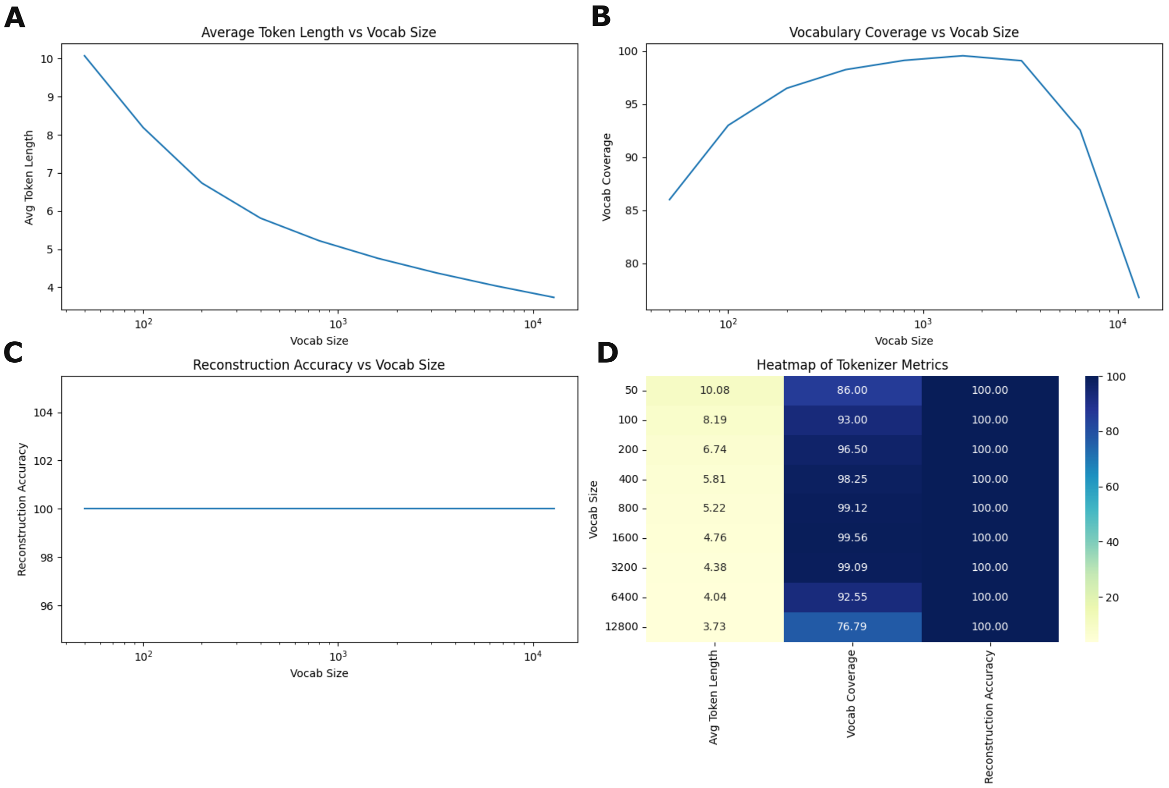

Figure S17: Training of tokenizer using TCR and epitope sequences, related to STAR Methods Evaluation metrics across varying vocabulary sizes. **a.** Average token length versus vocabulary size, showing the relationship between sequence tokenization granularity and vocabulary capacity (vocabulary sizes doubled incrementally from 50 to 12,800). **b.** Vocabulary coverage as a function of vocabulary size, demonstrates the proportion of sequences that can be successfully tokenized. **c.** Reconstruction accuracy versus vocabulary size, illustrating the model's ability to faithfully reconstruct original sequences. **d.** Composite heatmap visualization integrates all three metrics (average token length, vocabulary coverage, and reconstruction accuracy) across the range of vocabulary sizes, enabling simultaneous assessment of these key performance indicators.

Unpaired Peptide Dataset Sources			
**Type**	**NetMHCPan**	**MHCFlurry**	**SysteMHC**
Unique peptide	12,081,588	13,195,789	449,852
Unique class I peptide	9,147,682	13,178,655	442,746
**Merge of NetMHCPan, MHCFlurry, and SysteMHC**	**EpiNegSet (for training Robust Affinity Predictor)**	**EpiCandidateSet (for pseudo-labeling and training EpitopeGen)**	**Total**
Train	1,601,187		
Val			
Test	200,000		
Total	1,801,187	20,000,000	21,801,187
**Unpaired TCR Dataset Sources**			
**TCRdb**	**TCRNegSet (for training Robust Affinity Predictor)**	**TCRCandidateSet (for pseudo-labeling and training EpitopeGen)**	**Total**
Train	380,000		
Val			
Test	120,000		
Total	500,000	6,831,478	7,331,478

Table S1: Summary of the unpaired datasets of TCR and peptide sequences, related to Figure 1 The peptide datasets are from three sources: NetMHCPan v4.0[13], MHCFlurry v2.0[14], and SysteMHC[15], which were preprocessed, merged, and split into two: EpiNegSet and EpiCandidateSet. The unpaired TCRs were obtained from the TCRdb[16] dataset and were split into two: TCRNegSet and TCRCandidateSet.

$(n_{\text{max\_tcr}}, n_{\text{max\_epi}}) = (32, 100)$, **after ACF**	**Train**	**Val**	**Test**
Unique TCR	480,423	120,563	59,815
Unique epitope	71,028	37,876	25,332
Total rows	527,828	123,602	60,556
TCR-to-epitope ratio	6.76	3.18	2.36
$(n_{\text{max\_tcr}}, n_{\text{max\_epi}}) = (32, 100)$, **before ACF**	**Train**	**Val**	**Test**
Unique TCR	3,928,055	2,044,980	1,251,077
Unique epitope	1,037,034	710,506	546,014
Total rows	11,836,453	3,398,753	1,674,013
TCR-to-epitope ratio	3.79	2.88	2.29

Table S2: Summary of the pseudo-labeled datasets per split, related to Figure 1 Here, $n_{\text{max\_tcr}}$ denotes the maximum number of selected epitopes for a TCR and $n_{\text{max\_epi}}$ denotes the maximum occurrences of an epitope. The TCR-to-epitope ratio is the number of unique TCRs divided by the number of unique epitopes.

$n_{\text{max\_epi}}$	**Inf**	**800**	**400**	**200**	**100**
Total rows	224,526,336	81,575,641	50,091,251	29,489,056	16,909,219
Unique tcr	6,762,496	6,757,435	6,577,021	5,787,948	4,514,443
Unique epitope	1,437,906	1,354,151	1,298,733	1,226,493	1,138,726
TCR-to-epitope ratio	4.7	4.99	5.06	4.72	3.96
Unique epitope ratio	0.00640	0.0165	0.0259	0.0415	0.0673
Reduction ratio	-	0.636	0.777	0.869	0.925

Table S3: Summary of the pseudo-labeled datasets by $n_{\text{max\_tcr}}$, related to Figure 1 Here, $n_{\text{max\_epi}}$ denotes the maximum occurrences of an epitope, TCR-to-epitope ratio is given by the number of unique TCRs divided by the number of unique epitopes, unique epitope ratio is given by the number of unique epitopes divided by total rows, and reduction ratio is given by (224,526,336 - total rows)/224,526,336. The table highlights that the data before the Redundancy Filter contains a large number of redundant epitopes.

Public Paired Datasets					
	**IEDB**	**VDJdb**	**McPAS-TCR**	**PIRD**	**Total**
Unique TCR	86,519	6,131	10,081	5,174	98,460
Unique epitope	914	361	313	54	1,141
Total rows	91,201	6,479	12,879	5,498	116,057
TCR-to-epitope ratio	94.66	16.98	32.21	95.81	86.29
**Training Set**					
Unique TCR	61,393	4,334	7,290	3,644	71,263
Unique epitope	804	340	288	50	1,044
Total rows	63,840	4,535	9,015	3,848	81,238
TCR-to-epitope ratio	76.36	12.75	25.31	72.88	68.26
**Validation Set**					
Unique TCR	18,082	1,280	2,299	1,081	22,106
Unique epitope	512	204	185	43	694
Total rows	18,331	1,302	2,588	1,105	23,326
TCR-to-epitope ratio	35.32	6.27	12.43	25.14	31.85
**Test Set**					
Unique TCR	8,948	638	1,134	538	11,092
Unique epitope	388	146	125	36	518
Total rows	9,030	642	1,276	545	11,493
TCR-to-epitope ratio	23.06	4.37	9.07	14.94	21.41

Table S4: Summary of publicly available paired TCR and epitope datasets, related to Figure 1 Unique TCR and unique epitope denote the number of unique TCRs and epitopes in the dataset. Total rows denote the number of pairs included. TCR-to-epitope ratio is unique TCR divided by unique epitope. The Train:Val:Test was split into the 7:2:1 ratio. The datasets were used to train Robust Affinity Predictor. The train set was merged with the pseudo-labeled dataset to train EpitopeGen. The test set was used to assess the binding affinities and naturalness of the generated epitopes.

Glanville	Total (Test)
Unique TCR	1,966
Unique epitope	7
Total rows	4,120
TCR-to-epitope ratio	280.86
**MIRA**	**Total (Test)**
Unique TCR	136,463
Unique epitope	419
Total rows	154,320
TCR-to-epitope ratio	325.69

Table S5: Summary of external datasets, related to Figure 1 The external datasets are from two sources: the Glanville dataset published by Glanville et al.[8] and the MIRA dataset published by Nolan et al.[9] The datasets show a high TCR-to-epitope ratio, indicating relatively low diverse peptides relative to TCRs.

Protein	Amino Acid Sequence
TCR Alpha Chain Template	AQEVTQIPAALSVPEGENLVLNCSFTDSAIYNLQWFRQDPGK GLTSLLLIQSSQREQTSGRLNASLDKSSGRSTLYIAASQPGD SATYLCAVTNQAGTALIFGKGTTLSVSS
TCR Beta Chain Template	NAGVTQTPKFQVLKTGQSMTLQCSQDMNHEYMSWYRQDPGM GLRLIHYSVGAGITDQGEVPNGYNVSRSTTEDFPLRLLSAA PSQTSVYFCASSYSIRGSRGEQFFGPGTRLTVL
HLA-A*02:01 Sequence	MAVMAPRTLVLLLSGALALTQTWAGSHSMRYFFTSVSRPGRGEPRFIAV GYVDDTQFVRFDSDAASQRMEPRAPWIEQEGPEYWDGETRKVKAHSQTH RVDLGTLRGYYNQSEAGSHTVQRMYGCDVGSDWRFLRGYHQYAYDGKDY IALKEDLRSWTAADMAAQTTKHKWEAAHVAEQLRAYLEGTCVEWLRRYL ENGKETLQRTDAPKTHMTHHAVSDHEATLRCWALSFYPAEITLTWQRDG EDQTQDTELVETRPAGDGTFQKWAAVVVPSGQEQRYTCHVQHEGLPKPL TLRWEPSSQPTIPIVGIIAGLVLFGAVITGAVVAAVMWRRKSSDRKGGS YSQAASSDSAQGSDVSLTACKV

Table S6: Protein sequences used in molecular dynamics (MD) simulations, related to Figure 4 The table presents the amino acid sequences of three proteins used in MD simulations and subsequently processed through TCRmodel2[17] for structure prediction. The TCR alpha chain and the HLA-A02:01 sequences remained constant throughout the simulations. To evaluate structural stability, the CDR3β region of the TCR beta chain template was substituted with the generated epitope sequences. HLA-A02:01 was chosen for this analysis because it is a predominant human HLA allele.

Method	Total clusters	Non-singleton clusters	Coverage	Median size (non-sing.)	Mean subj. incidence	Enriched per fold
GLIPH2	12,864	836	99.7%	2	3.1	4 ± 2
TCRdist3	11,908	896	97.1%	2	3.8	3 ± 2
DeepTCR	139	139	100%	93	50.3	1 ± 1
iSMART	157	157	2.5%	2	2.3	1 ± 0

Table S7: Clustering characteristics across TCR grouping methods, related to Figure 6 Subject incidence and enrichment statistics are computed over non-singleton clusters ($|G_c| \geq 2$). Enrichment statistics are averaged across 5 cross-validation folds.

Method	Feature source	Model	AUROC $\pm$ 95% CI	AUPRC	F1
Setting 1: Beta-binomial framework					
Emerson[18]	Public TCRs	Beta-bin	0.557 ± 0.090	0.378	0.076
GLIPH2	Motif groups	Beta-bin	0.658 ± 0.098	0.467	0.258
TCRdist3	Distance clusters	Beta-bin	0.581 ± 0.099	0.346	0.149
DeepTCR	VAE clusters	Beta-bin	0.573 ± 0.162	0.350	0.240
iSMART	Alignment clusters	Beta-bin	0.634 ± 0.095	0.421	0.143
Setting 2: Logistic regression, TCR-derived features only					
GLIPH2	Motif groups	LogReg	0.514 ± 0.107	0.385	0.390
TCRdist3	Distance clusters	LogReg	0.621 ± 0.091	0.384	0.321
DeepTCR	VAE clusters	LogReg	0.639 ± 0.094	0.492	0.405
iSMART	Alignment clusters	LogReg	0.484 ± 0.091	0.311	0.456
EpitopeGen	Protein targets	LogReg	0.612 ± 0.095	0.398	**0.539**
Setting 3: Logistic regression with gene expression					
GLIPH2	Groups + GEX	LogReg	0.731 ± 0.095	0.615	**0.619**
TCRdist3	Clusters + GEX	LogReg	0.641 ± 0.104	0.453	0.557
DeepTCR	Clusters + GEX	LogReg	0.650 ± 0.096	0.483	0.487
iSMART	Clusters + GEX	LogReg	0.686 ± 0.106	0.576	0.560
EpitopeGen	Proteins + GEX	LogReg	**0.762** ± 0.087	**0.620**	0.540

Table S8: Classification performance for COVID-19 severity prediction, related to Figure 6 mild vs. severe, $N_{\text{subjects}} = 158$, $N_{\text{CDR3}\beta} = 15{,}346$, 5-fold stratified CV, 95% CIs via 1,000 bootstrap resamples. Beta-bin: beta-binomial model; LogReg: L1-regularized logistic regression.

Category	Classification Criteria
Virus	**Families:** Viridae suffixes<br>**Special markers:** HIV, immunodeficiency, SARS, coronavirus<br>**Retroviral markers:** Retroviridae, retrovirus<br>**Phage families:** Caudovirales, Myoviridae, Siphoviridae, Podoviridae<br>**Exclusions:** Bacteriophage, provirus
Parasite	**Genera:** *Plasmodium*, *Trypanosoma*, *Leishmania*, *Toxoplasma*, *Cryptosporidium*, *Giardia*, *Entamoeba*, *Trichomonas*, *Babesia*, *Theileria*, *Schistosoma*, *Fasciola*, *Taenia*, *Echinococcus*, and others<br>**Families:** Plasmodiidae, Trypanosomatidae, Toxoplasmatidae, Cryptosporidiidae, Giardiidae, Entamoebidae, Trichomonadidae, and others<br>**Phyla:** Apicomplexa, Platyhelminthes, Nematoda, Arthropoda<br>**Protozoan phyla:** Apicomplexa, Ciliophora, Euglenozoa, Amoebozoa, Microsporidia
Fungi	**Kingdom markers:** fungi, mycota
Bacteria	**Superkingdom:** Bacteria
Archaea	**Superkingdom:** Archaea

Table S9: Taxonomic classification criteria for antigen categories, related to STAR Methods The classification criteria for each category used to categorize the species according to lineage information. Bacteriophages were excluded because they infect bacteria and proviruses were excluded as they behave like self-antigens.

References

1. Francis, J. M., Leistritz-Edwards, D., Dunn, A., Tarr, C., Lehman, J., Dempsey, C., Hamel, A., Rayon, V., Liu, G., Wang, Y., et al. (2022). Allelic variation in class I HLA determines CD8+ T cell repertoire shape and cross-reactive memory responses to SARS-CoV-2. Science Immunology *7*, eabk3070.

2. Shugay, M., Bagaev, D. V., Zvyagin, I. V., Vroomans, R. M., Crawford, J. C., Dolton, G., Komech, E. A., Sycheva, A. L., Koneva, A. E., Egorov, E. S., et al. (2018). VDJdb: a curated database of T-cell receptor sequences with known antigen specificity. en. Nucleic Acids Res *46*, D419–D427.

3. Vita, R., Mahajan, S., Overton, J. A., Dhanda, S. K., Martini, S., Cantrell, J. R., Wheeler, D. K., Sette, A., Sette, A., Peters, B., et al. (2019). The Immune Epitope Database (IEDB): 2018 update. Nucleic Acids Research *47*.

4. Zhang, W., Wang, L., Liu, K., Wei, X., Yang, K., Du, W., Wang, S., Guo, N., Ma, C., Luo, L., et al. (2019). PIRD: Pan Immune Repertoire Database. Bioinformatics *36*, 897–903.

5. Tickotsky, N., Sagiv, T., Prilusky, J., Shifrut, E., and Friedman, N. (2017). McPAS-TCR: a manually curated catalogue of pathology-associated T cell receptor sequences. en. Bioinformatics *33*, 2924–2929.

6. Meynard-Piganeau, B., Feinauer, C., Weigt, M., Walczak, A. M., and Mora, T. (2024). TULIP: A transformer-based unsupervised language model for interacting peptides and T cell receptors that generalizes to unseen epitopes. Proceedings of the National Academy of Sciences *121*, e2316401121. 10.1073/pnas.2316401121. eprint: https://www.pnas.org/doi/pdf/10.1073/pnas.2316401121.

7. Henikoff, S. and Henikoff, J. G. (1992). Amino acid substitution matrices from protein blocks. en. Proc Natl Acad Sci U S A *89*, 10915–10919.

8. Glanville, J., Huang, H., Nau, A., Hatton, O., Wagar, L. E., Rubelt, F., Ji, X., Han, A., Krams, S. M., Pettus, C., et al. (2017). Identifying specificity groups in the T cell receptor repertoire. Nature *547*, 94–98.

9. Nolan, S., Vignali, M., Klinger, M., Dines, J. N., Kaplan, I. M., Svejnoha, E., Craft, T., Boland, K., Pesesky, M., Gittelman, R. M., et al. (2020). “A large-scale database of T-cell receptor beta (TCRβ) sequences and binding associations from natural and synthetic exposure to SARS-CoV-2”. en.

10. Huang, H., Wang, C., Rubelt, F., Scriba, T. J., and Davis, M. M. (2020). Analyzing the Mycobacterium tuberculosis immune response by T-cell receptor clustering with GLIPH2 and genome-wide antigen screening. Nature Biotechnology *38*, 1194–1202.

11. Leaver-Fay, A., Tyka, M., Lewis, S. M., Lange, O. F., Thompson, J., Jacak, R., Kaufman, K., Renfrew, P. D., Smith, C. A., Sheffler, W., et al. (2011). ROSETTA3: an object-oriented software suite for the simulation and design of macromolecules. en. Methods Enzymol *487*, 545–574.

12. Stranges, P. B. and Kuhlman, B. (2012). A comparison of successful and failed protein interface designs highlights the challenges of designing buried hydrogen bonds. en. Protein Sci *22*, 74–82.

13. Jurtz, V., Paul, S., Andreatta, M., Marcatili, P., Peters, B., and Nielsen, M. (2017). NetMHCpan-4.0: Improved Peptide-MHC Class I Interaction Predictions Integrating Eluted Ligand and Peptide Binding Affinity Data. en. J Immunol *199*, 3360–3368.

14. O'Donnell, T. J., Rubinsteyn, A., and Laserson, U. (2020). MHCflurry 2.0: Improved Pan-Allele Prediction of MHC Class I-Presented Peptides by Incorporating Antigen Processing. Cell Systems *11*, 42–48.e7.

15. Huang, X., Gan, Z., Cui, H., Lan, T., Liu, Y., Caron, E., and Shao, W. (2023). The SysteMHC Atlas v2.0, an updated resource for mass spectrometry-based immunopeptidomics. Nucleic Acids Research *52*, D1062–D1071.

16. Chen, S.-Y., Yue, T., Lei, Q., and Guo, A.-Y. (2021). TCRdb: a comprehensive database for T-cell receptor sequences with powerful search function. en. Nucleic Acids Res *49*, D468–D474.

17. Yin, R., Ribeiro-Filho, H. V., Lin, V., Gowthaman, R., Cheung, M., and Pierce, B. G. (2023). TCRmodel2: high-resolution modeling of T cell receptor recognition using deep learning. en. Nucleic Acids Res *51*, W569–W576.

18. Emerson, R. O., DeWitt, W. S., Vignali, M., Gravley, J., Hu, J. K., Osborne, E. J., Desmarais, C., Klinger, M., Carlson, C. S., Hansen, J. A., et al. (2017). Immunosequencing identifies signatures of cytomegalovirus exposure history and HLA-mediated effects on the T cell repertoire. Nature Genetics *49*, 659–665.
